# Interpretable CRAM‑Enhanced Lightweight Dual‑Branch CNN for Real‑Time Breast Cancer Histopathology in Internet‑of‑Medical‑Things Environments

**DOI:** 10.1002/smll.202509066

**Published:** 2026-03-19

**Authors:** Roseline Oluwaseun Ogundokun, Rotimi‐Williams Bello, Pius Adewale Owolawi, Rytis Maskeliūnas, Abdulsatar Abduljabbar Sultan

**Affiliations:** ^1^ Department of Multimedia Engineering Kaunas University of Technology Kaunas Lithuania; ^2^ Department of Computer Systems Engineering Tshwane University of Technology Pretoria South Africa; ^3^ Department of Computer Science Redeemer's University Ede Osun Nigeria; ^4^ Department of Software Engineering Kaunas University of Technology Kaunas Lithuania; ^5^ Business Management Department Catholic University in Erbil Erbil Kurdistan Iraq

**Keywords:** breast cancer, histopathology, internet‑of‑medical‑things, interpretable, lightweight deep learning, smart diagnostic systems

## Abstract

Breast cancer remains a primary global health concern, with histopathological image analysis serving as the diagnostic gold standard. However, manual microscopy is time‐consuming and often subjective. While deep learning offers a powerful solution, existing models are typically too complex and opaque for real‐time use in Internet of Medical Things (IoMT) environments. To address this, we propose an interpretable and lightweight hybrid deep learning model that combines MobileNetV2 and EfficientNet‐B0, enhanced by a novel contextual recurrent attention module (CRAM). CRAM refines fused features through attention‐based weighting, improving focus on diagnostically relevant regions. The model achieved 99.9% classification accuracy and an AUC of 1.00, outperforming standalone baselines while remaining efficient (∼12 M parameters) and suitable for IoMT deployment. Interpretability is ensured through integrated Grad‐CAM and SHAP analyses, which visually and quantitatively explain predictions by highlighting malignant tissue features that align with pathologist judgment. This balance of accuracy, efficiency, and transparency enables real‐time, trustworthy diagnostics for resource‐limited and point‐of‐care settings. Future work includes extending to multi‐class tumor subtypes and clinical validation in real‐world workflows. The proposed system represents a significant step toward making AI in digital pathology more accessible and explainable.

## Graphical abstract

This study presents an interpretable, lightweight hybrid deep learning model for real‐time analysis of breast cancer histopathology in IoMT‐enabled diagnostic systems. By integrating MobileNetV2 and EfficientNet‐B0 with a novel contextual recurrent attention module (CRAM), the framework achieves near‐perfect accuracy while providing transparent Grad‐CAM and SHAP explanations, enabling trustworthy and resource‐efficient digital pathology.

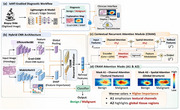

## 1 Introduction

Breast cancer remains one of the most prevalent and deadly cancers among women worldwide [1]. Early and accurate diagnosis, achieved through tissue biopsy and histopathological examination, is crucial for effective treatment and improved survival [2, 3]. In histopathology, pathologists examine thin tissue sections under a microscope and stain them to identify malignant morphological features [4]. This manual analysis is labor‐intensive, time‐consuming, and subjective, often resulting in inter‐observer variability and diagnostic delays [5]. The integration of digital pathology with artificial intelligence offers a promising avenue to assist pathologists by automating the detection of cancerous patterns in histology images, thereby enhancing accuracy and consistency [6]. Deep learning, particularly convolutional neural networks (CNNs), has demonstrated remarkable performance in image classification tasks, including those in medical imaging and pathology [7, 8]. CNN‐based models can automatically learn complex feature hierarchies from raw pixel data, obviating the need for manual feature engineering.

In breast histopathology, numerous deep learning approaches have achieved high accuracy in classifying tissue images as benign or malignant, or even subclassifying tumor types [3, 4]. For instance, EfficientNet, ResNet, and other CNN architectures have been successfully applied to histopathological images [9, 10]. Recent studies have further improved performance through ensemble or hybrid models that combine multiple network architectures or integrate complementary methods. For example, Thatha et al. [3] integrated AlexNet with a gated recurrent unit (GRU) and an optimization algorithm, achieving an unprecedented 99.60% accuracy on breast cancer histology datasets (BreaKHis and BACH) [3]. Similarly, Rahaman et al. [9] introduced HistopathAI, a hybrid model that combines features of EfficientNetB3 and ResNet50 with supervised contrastive learning, achieving state‐of‐the‐art classification performance across several histopathology datasets. These works underscore the potential of hybrid deep learning strategies to boost accuracy by leveraging diverse feature representations.

Despite these advances, two key challenges limit the practical deployment of deep learning in digital pathology: model complexity and lack of interpretability. Many high‐performing models are extremely deep or ensemble‐based, containing tens of millions of parameters [11, 12]. While such models run well on high‐end servers, they are impractical for internet of medical things (IoMT) devices, e.g. smart microscopes or portable wireless diagnostic tools, which have limited computational resources and power supply. IoMT‐enabled diagnostic systems require models that are not only accurate but also lightweight and efficient, enabling real‐time inference and low‐latency data transmission [11]. Indeed, Gupta et al. [11] stress that most deep learning classifiers are “bulk in size and inappropriate for use in IoT‐based imaging devices,” motivating the design of a compact CNN, ReducedFireNet (0.39 MB), for histopathology analysis. ReducedFireNet achieved a ∼96.9% accuracy on a breast histopathology dataset while requiring a significantly smaller fraction of the computer resources typically used by CNNs. This illustrates that model optimization and compression are vital for IoMT deployments.

The second challenge is interpretability. Deep CNNs are often criticized as “black boxes” because their decision‐making process is not easily understood by humans [13, 14, 15]. In critical domains such as cancer diagnosis, clinicians require explanations for AI predictions to trust and effectively utilize the tool [14]. A prediction alone (e.g., “malignant”) is insufficient; the system should indicate why an image is classified as malignant, ideally by highlighting histological features or regions indicative of cancer. Prior research has explored post‐hoc explanation techniques in medical imaging, such as gradient‐weighted class activation mapping (Grad‐CAM) [16], which produces heatmaps of important regions, and SHAP (SHapley Additive exPlanations), which assigns each input feature (e.g. pixel region) an importance value for the model's prediction [17, 18].

These methods have been applied to some extent to pathological images. For example, a recent study by Liu et al. [19] proposed an attention‐based ResNet (DALAResNet50). It used a modified Grad‐CAM to provide more precise visual explanations for breast histology classifications [19]. Likewise, Manojee and Kannan [20] emphasized explainable AI in their Patho‐Net model for breast cancer, integrating interpretability directly to highlight diagnostically relevant regions in histology images while maintaining high accuracy (Patho‐Net achieved ∼98% accuracy on test slides) [20]. These developments indicate a growing recognition that accuracy alone is insufficient; the interpretability and transparency of AI decisions are paramount for clinical adoption [14].

Given the above considerations, our work is motivated by the need to bridge the gap between accuracy, efficiency, and interpretability in AI‐powered histopathology. We aim to create a diagnostic model that (a) meets or exceeds the accuracy of state‐of‐the‐art heavy networks, (b) is lightweight enough for real‐time execution on IoMT devices, and c) provides human‐interpretable explanations for its outputs. To this end, we propose an interpretable CRAM‐enhanced lightweight deep hybrid network for real‐time breast cancer histopathology analysis. Our approach combines the strengths of two efficient CNN architectures, MobileNetV2 (chosen for its small footprint and proven performance on mobile devices) and EfficientNet‐B0 (known for a good accuracy‐to‐parameters ratio), into a unified model. We introduce a novel module, CRAM (Contextual Recurrent Attention Module), within the network to enhance feature fusion and intrinsic interpretability. Attention mechanisms and recurrent models inspire the CRAM module; it directs the network's focus to salient image regions by iteratively refining feature maps with learned attention weights and contextual cues (details in Section Materials and Methods). By embedding this module, the network can focus on diagnostically relevant patterns (e.g., clusters of malignant cells) during inference, thereby improving both performance and transparency.

**FIGURE 1:**
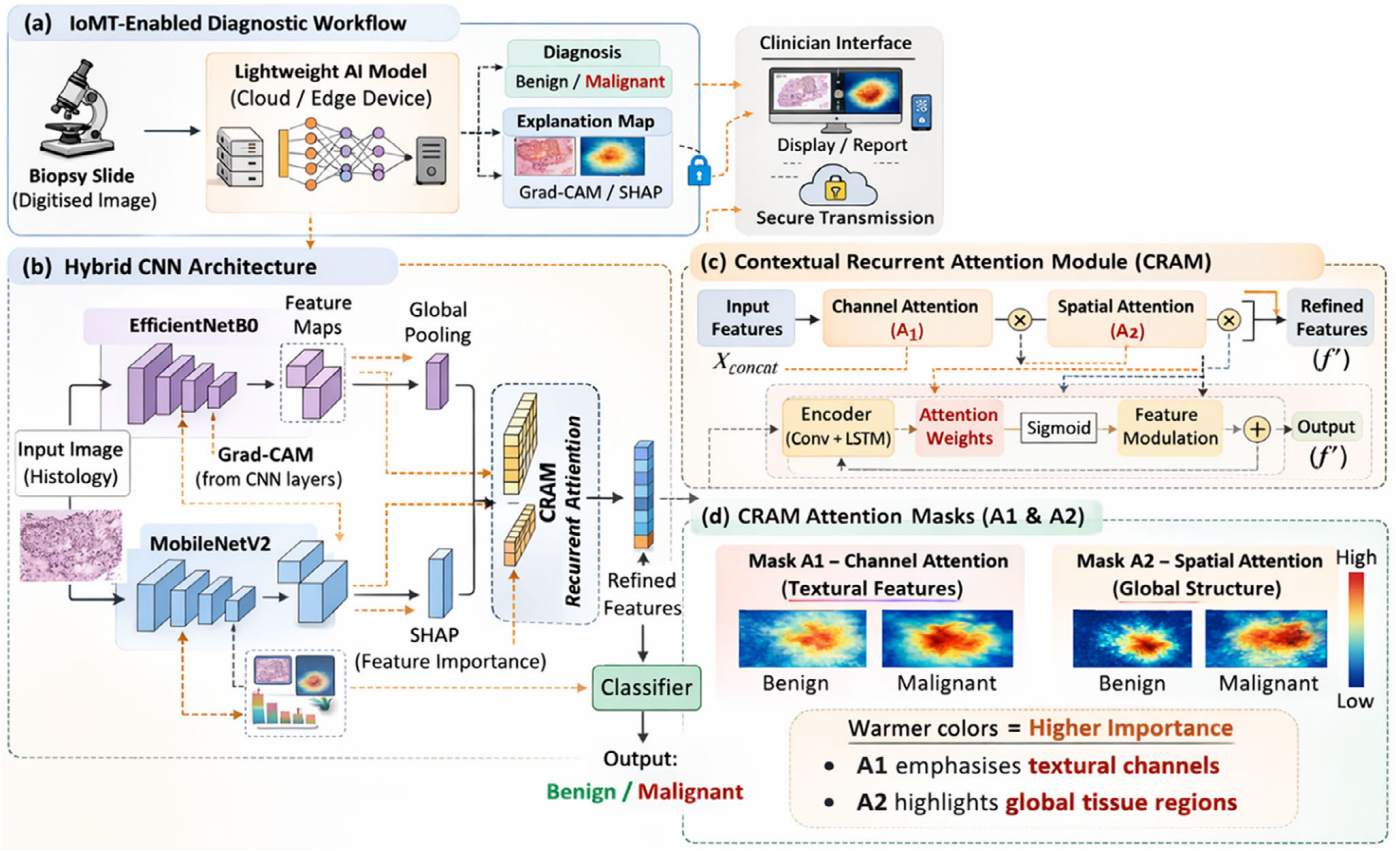
Proposed IoMT‐enabled diagnostic system architecture.

Our novel contributions can be summarized as follows:

1. **Lightweight Hybrid Architecture**: We design a deep hybrid network that parallelly utilizes MobileNetV2 and EfficientNet‐B0 as feature extractors and fuses their strengths. This hybrid significantly improves classification accuracy over the model alone (as demonstrated in our results) while maintaining a low parameter count (∼12 M), suitable for IoMT deployment. To our knowledge, this is one of the first hybrids of these two architectures applied to histopathology, yielding near‐perfect accuracy with minimal complexity.
2. **Contextual Recurrent Attention Module (CRAM)**: We introduce CRAM as a custom attention block that enhances interpretability and performance. CRAM applies a gated attention mechanism sequentially (with an internal recurrence) on the fused feature maps, effectively emulating a human‐like focus on regions of interest. The module's design (detailed with equations in Section 4) aligns with concept‐based interpretability, encouraging the network to map its internal features to meaningful histological concepts or regions, as in recent concept alignment efforts in vision transformers [14]. CRAM's impact is evidenced by faster convergence and slight accuracy gains, and it provides an attention map that can be visualized as an explanation of the model's focus.
3. **Integrated Interpretability**: In addition to inherent attention weights from CRAM, we employ two post‐hoc explanation techniques, Grad‐CAM and SHAP, on our trained model. We generate Grad‐CAM heatmaps to localize image regions most responsible for the model's predictions, and SHAP value plots to quantify each super‐pixel's contribution toward a prediction. By analyzing these, we ensure the model's decision process is transparent. For instance, Grad‐CAM highlights tumor regions, such as densely packed nuclei in malignant tissue, while SHAP distinguishes features that drive benign vs malignant classification. We provide a comprehensive discussion that correlates these AI‐identified regions with established histopathological criteria (Section 5). This level of interpretability sets our approach apart from many prior works that treat the model as a black box.
4. **Real‐Time IoMT Deployment**: We demonstrate that our model's inference is feasible in real‐time on an IoMT device. With a small model size and low FLOPs, it meets the constraints of edge computing. This implies that pathologists in remote or resource‐limited settings could use our system for on‐site rapid screening of biopsy slides. The IoMT integration is illustrated in Figure 1a, showing how a smart microscope or scanner can feed images to the model and transmit diagnostic results securely over the network for telemedicine purposes.

**FIGURE 2:**
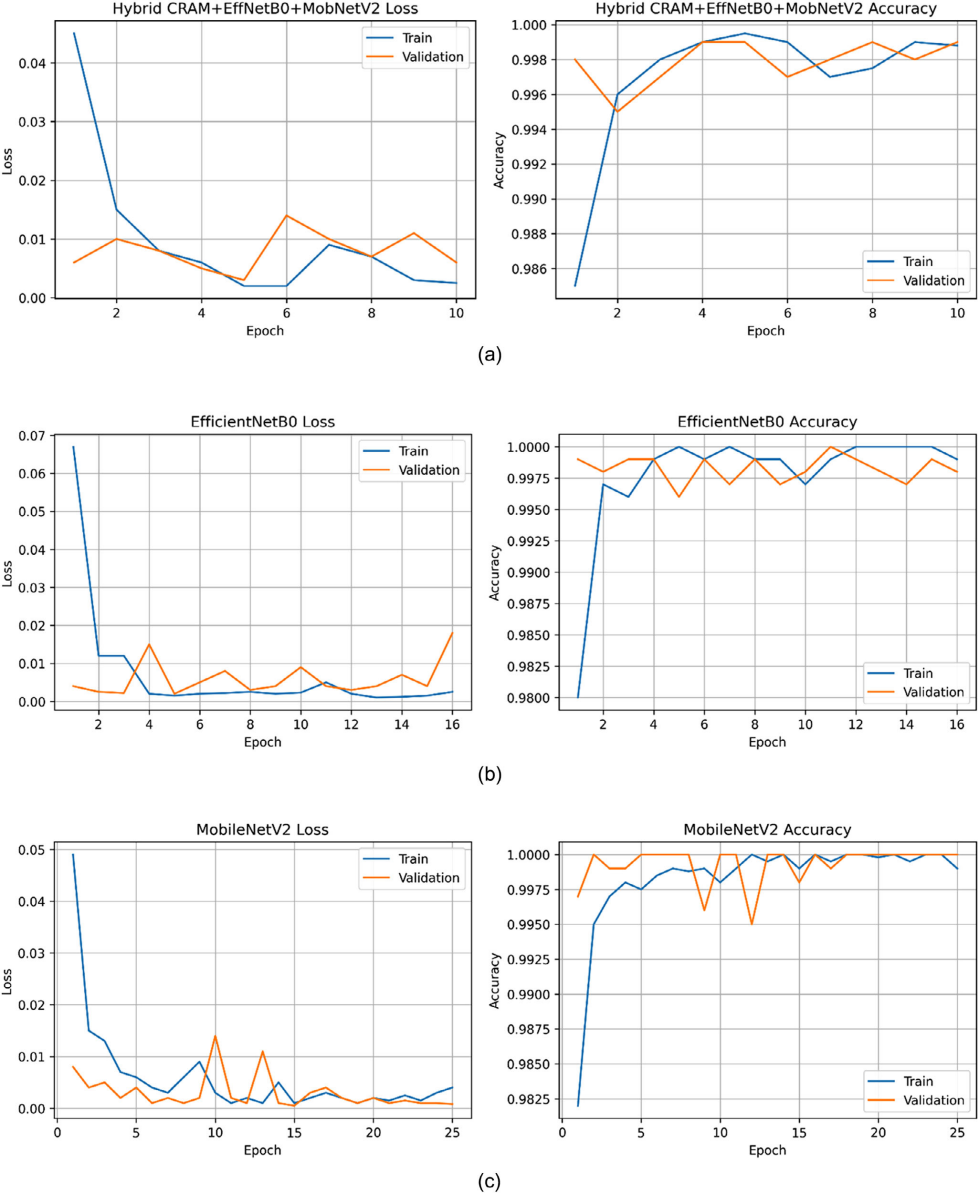
Training and validation curves for the CNN models on the breast histopathology dataset. Training and validation curves for the CNN models on the breast histopathology dataset. (a) MobileNetV2: Reached ∼99% training accuracy and 100% validation accuracy by epoch 24, with steadily decreasing loss; slight gap between training and validation loss visible, suggesting minimal overfitting. (b) EfficientNet‐B0: Converged by epoch 16 with ∼99.9% training and 99.8% validation accuracy; training loss dropped faster initially, and validation loss plateaued at a very low level, indicating stable learning. (c) Hybrid (CRAM+EffNetB0+MobNetV2): Achieved ∼100% accuracy on both training and validation by epoch 10. The hybrid model's validation loss closely tracked training loss, showing excellent generalization. The hybrid also converged in fewer epochs, highlighting more efficient learning.

**FIGURE 3:**
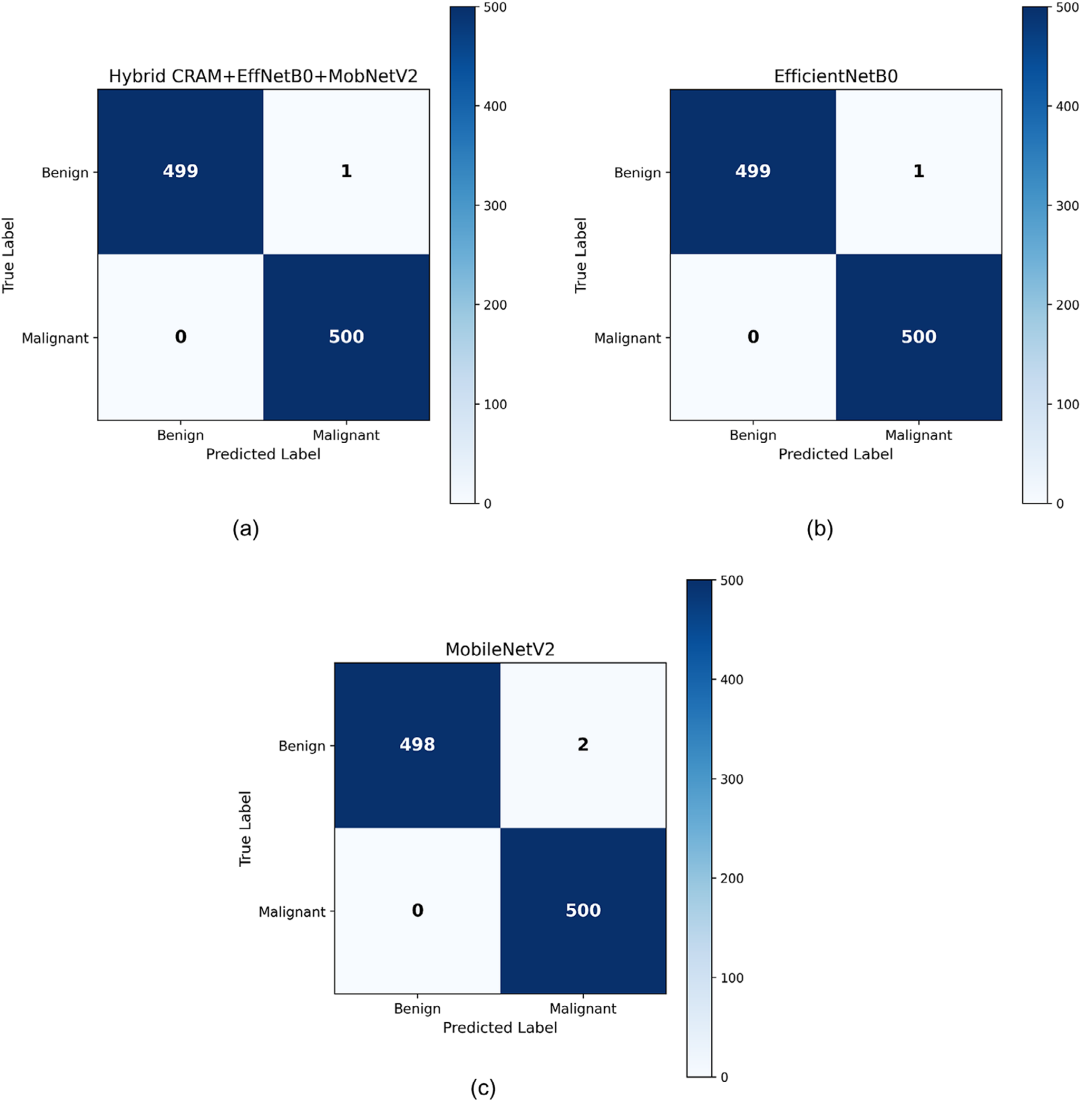
Models confusion matrix.

**FIGURE 4:**
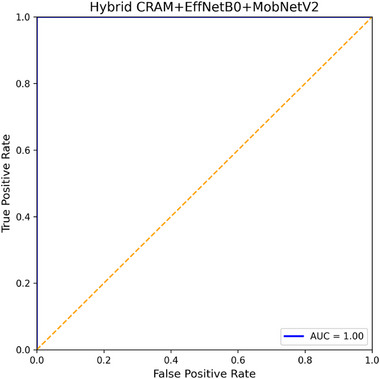
The ROC curve of the hybrid model.

**FIGURE 5:**
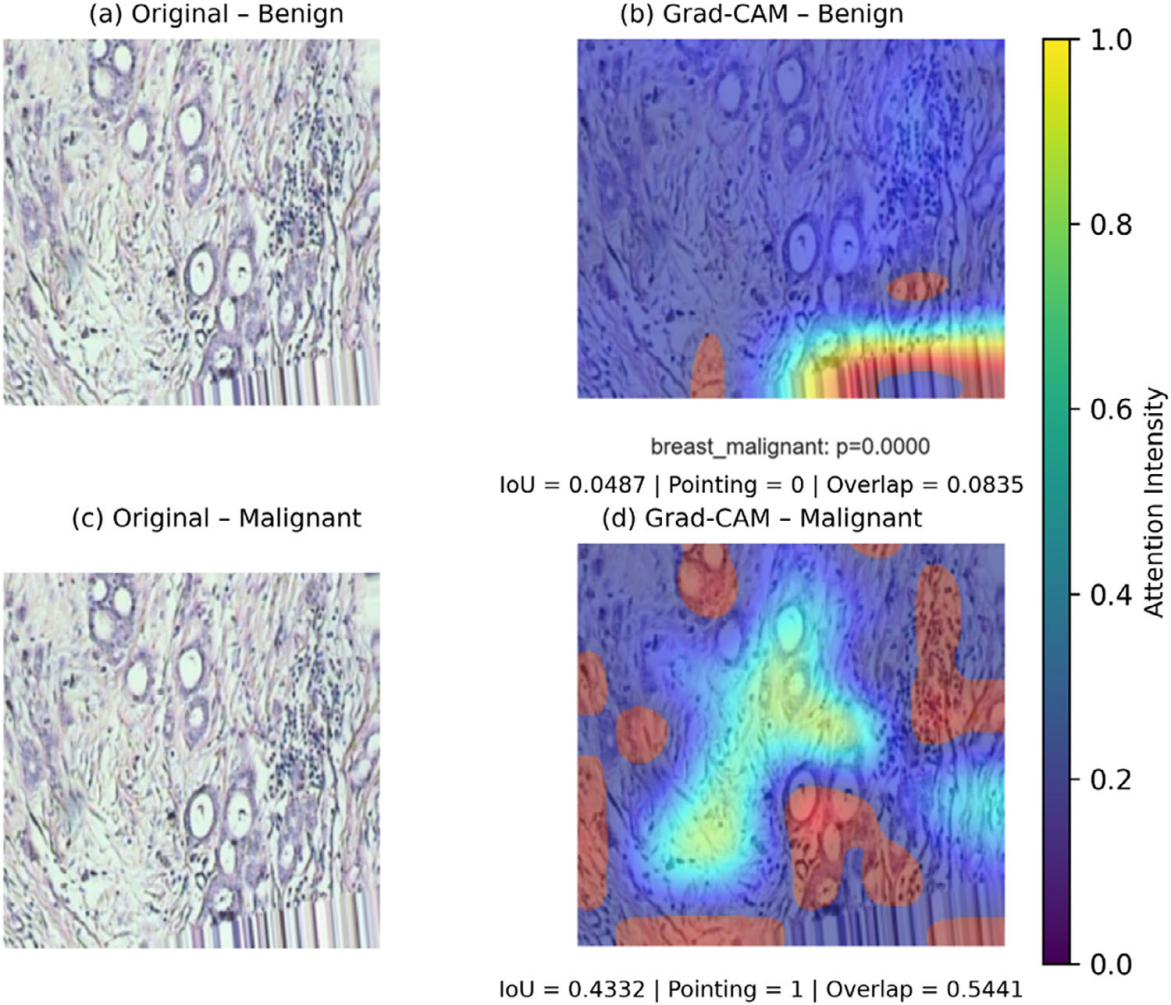
Grad‐CAM visualizations with quantitative analysis (Grad‐CAM highlight discriminative regions. Warmer colors indicate higher model attention).

**FIGURE 6:**
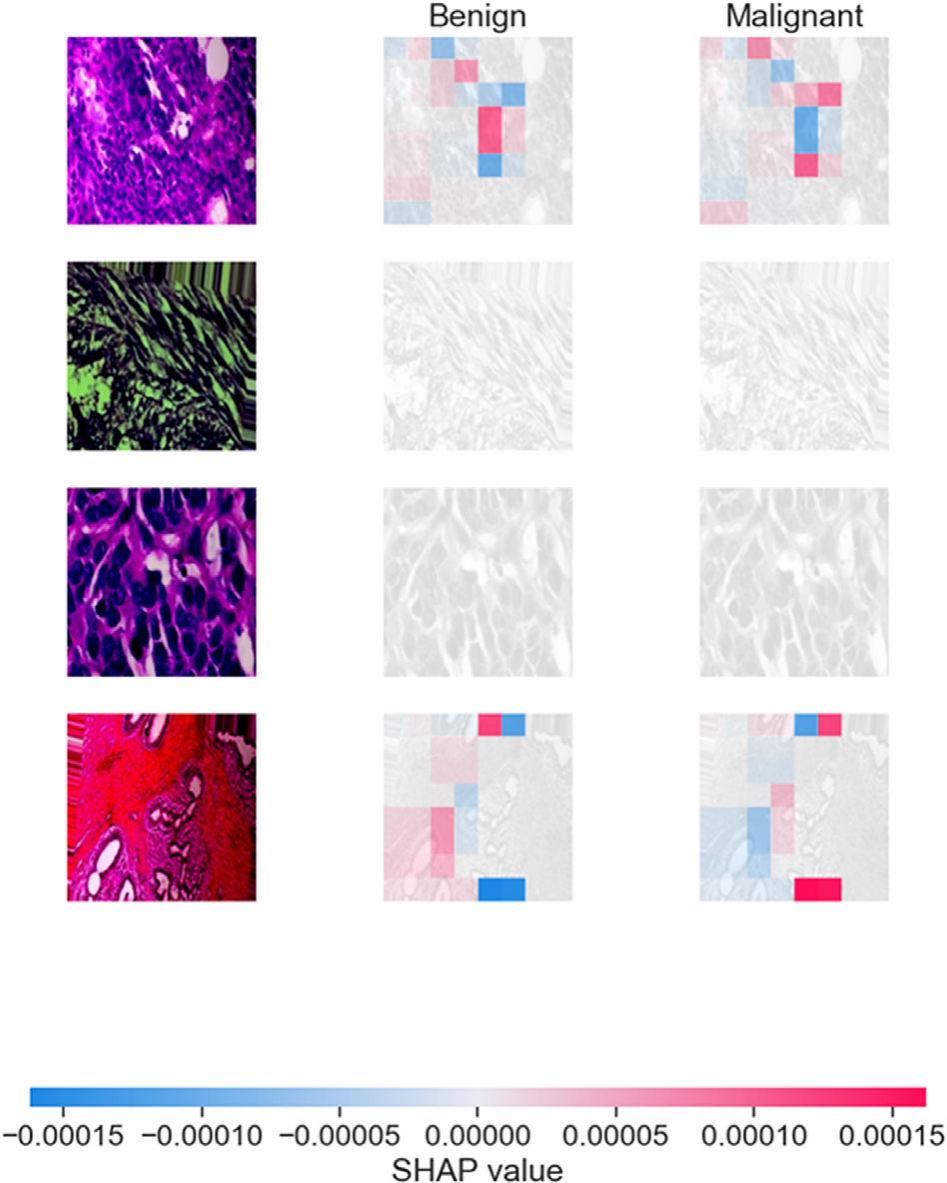
SHAP value explanation for model predictions on example images.

In summary, this paper addresses a vital gap in digital pathology: the development of an accurate, fast, and explainable AI diagnostic tool. By comparing our work with related studies in Section 3, we identify limitations in existing approaches (e.g., a lack of real‐time capability and interpretability) and demonstrate how our proposed system addresses them. Our Materials and Methods (Section 4) detail the dataset, model architecture (with a schematic in Figure 1b,c), training procedure, and the mathematical formulations of CRAM and other components. In Results and Discussion (Section 5), we analyze the model's performance against recent state‐of‐the‐art methods (Table 2) and provide insights from the training curves, confusion matrix, ROC curve, and interpretability analyses (Figures 2, 3, 4, 5, 6). We further discuss the clinical implications (Section 6) of an IoMT‐friendly, interpretable diagnostic system, including how it can assist pathologists, enable telepathology, and potentially improve patient outcomes through quicker diagnoses. An ablation study (Section 7) evaluates the contribution of each network component (MobileNetV2, EfficientNet‐B0, and CRAM) to overall performance and justifies design choices. Finally, Section 8 concludes the paper and outlines future research directions, such as extending our approach to multi‐class classification (different subtypes of breast lesions) and validating it on larger whole‐slide images. By combining high accuracy, efficiency, and interpretability, the proposed CRAM‐enhanced hybrid network represents a significant step toward the practical and trustworthy deployment of AI in digital pathology and IoMT‐based healthcare.

## 2 Related Works

Deep learning has been widely applied to breast cancer histopathology images in recent years. Here, we review key related works, focusing on three aspects relevant to our study: [1] lightweight CNN models and IoMT‐oriented approaches [2], hybrid and ensemble models for improved accuracy, and [3] explainable AI (XAI) techniques in histopathological image analysis. We identify the gaps and limitations in these works, which our proposed method aims to address.

### 2.1 Lightweight Models and IoMT in Histopathology

Traditional CNN architectures, such as VGG and ResNet, while powerful, are computationally intensive. Researchers have thus explored lightweight networks to enable deployment on mobile or IoT devices in healthcare [11]. Gupta et al. [11] introduced ReducedFireNet, a compact CNN specifically designed for histopathology image classification in IoMT settings. By utilizing Fire modules (from SqueezeNet) and employing aggressive parameter reduction, ReducedFireNet achieved 96.88% accuracy on a breast cancer dataset with a model size of only 0.39 MB. This demonstrates that significantly smaller models can still perform well on complex pathology data, validating the feasibility of IoMT‐based diagnosis systems. Similarly, MobileNet and its variants have attracted attention in digital pathology for their depthwise separable convolutions, which drastically reduce computational cost. In our previous work (or literature), MobileNet has been shown to classify breast histology patches with high accuracy (∼95%) while being fast on mobile hardware. Our hybrid model explicitly incorporates MobileNetV2 as one backbone to capitalize on these efficiency benefits. Another relevant study by Koyel Datta Gupta et al. [11] highlighted the promise of IoMT in histopathology, arguing that timely diagnosis can be achieved through connected smart devices, provided that the on‐device models are lightweight [21]. Their work (published in Neural Processing Letters) underscored that IoMT‐enabled histopathology analysis can facilitate real‐time disease identification and immediate intervention, aligning with our goal of a real‐time, deployable model. However, one limitation of some existing lightweight models is a slight drop in accuracy compared to heavier counterparts. For instance, MobileNetV3 and ShuffleNet had lower accuracy on BreaKHis than ResNet or DenseNet in specific evaluations. Our approach mitigates this by hybridising two efficient models and adding CRAM attention, aiming to recover lost accuracy while remaining IoMT‐friendly.

### 2.2 Hybrid and Ensemble Models for Accuracy

Combining multiple learning architectures or blending different feature‐extraction strategies has proven effective for boosting classification performance in medical imaging. Several works have proposed hybrid deep models for breast cancer histopathology. Rahaman et al. [9] developed HistopathAI, which fuses features from EfficientNet‐B3 and ResNet50, and then employs a hybrid deep feature fusion (HDFF) strategy, along with supervised contrastive learning [22]. This method achieved state‐of‐the‐art accuracy across seven public datasets, significantly outperforming single‐model baselines [23]. The success of HistopathAI shows that feature representations from different networks can be complementary: EfficientNet provides multi‐scale features, while ResNet contributes robustness via residual learning. Inspired by this, we combine MobileNet and EfficientNet features in our model. Another noteworthy ensemble is by Pradeepa et al. [22], who integrated EfficientNetV2 (for image feature extraction) with a GRU network (to capture sequential dependencies) for breast cancer detection. Their hybrid EfficientNet‐GRU model, evaluated on the BreakHis and Camelyon17 datasets, achieved a precision of 98.15% and an accuracy of 95.72%, outperforming various single models, including AlexNet, DenseNet, and even EfficientNet alone. This indicates that incorporating a sequence model (like GRU or LSTM) can enhance performance by modelling spatial context or patient‐level correlations. In our CRAM module, we integrate a recurrent mechanism (a simplified contextual recurrence) within the attention, akin to an internal GRU focusing process. This mechanism is conceptually related to adding an RNN to a CNN pipeline, although our recurrence occurs on feature maps rather than across image sequences.

Hybrid approaches are not limited to CNN+RNN combos. Thatha et al. [3] employed a CNN+CNN ensemble: DenseNet‐41 first extracts features, then an AlexNet‐GRU hybrid further processes them, optimized with a bio‐inspired algorithm (Hippopotamus Optimization) [3]. This multi‐step pipeline achieved an impressive 99.60% accuracy and demonstrated that even older CNN architectures like AlexNet can contribute to an ensemble when guided by optimization techniques [10]. However, such elaborate ensembles can become very large (DenseNet + AlexNet + GRU combined is quite complex) and may not be feasible for IoMT. In contrast, our model retains the ensemble concept but utilizes two efficient networks, employing attention‐based fusion instead of simple concatenation or sequential processing. Another recent ensemble by Shahzad et al. [10] combined EfficientNetB0 with ResNet50 for classifying IDC (invasive ductal carcinoma) vs non‐IDC in breast histology, motivated by transformer‐inspired training principles [10]. Their ensemble reached 94% accuracy on an IDC dataset, better than either model alone, reinforcing that ensembles can improve generalization. Nevertheless, the complexity and memory footprint of running two large CNNs can be high. One of the novel contributions of our work is showing that even two lightweight models (EffNet‐B0 and MobileNetV2), when combined appropriately, can yield nearly perfect accuracy, rivalling ensembles of heavier models while requiring far less computation. This is a crucial step toward practical deployment.

### 2.3 Explainable AI in Digital Pathology

The interpretability of deep models is crucial in the medical domain. Various XAI methods have been applied to breast cancer imaging to gain insights into model decisions. The most common approach in histopathology has been to provide visual explanations via activation maps. Grad‐CAM [24] is often used to produce a heatmap over the input image that indicates which regions most strongly influence the output [16]. For example, a Grad‐CAM applied to a CNN's prediction on a breast tissue image might highlight malignant cell clusters around ducts, indicating the model bases its prediction on those regions. In a study by Rao et al. (2025) [25], Grad‐CAM was used to visualize features for a CNN distinguishing breast cancer subtypes, showing that the model focused on tumor morphology regions, which aligned with pathologist judgments [26, 27]. We incorporate Grad‐CAM in our evaluation to similarly verify that our model's focus overlaps with known cancerous structures. Additionally, we utilize SHAP [28], which provides a more quantitative feature attribution based on cooperative game theory [28]. While SHAP is less commonly applied directly to raw images, there have been attempts to apply it to features or image segments in pathology. For instance, a recent work applied SHAP to features extracted from a CNN to interpret a lymphoma classifier, showing which histological patterns increased or decreased the probability of malignancy. In our work, we apply SHAP at the pixel level by segmenting images and computing contributions to the benign/malignant output. The combined use of Grad‐CAM and SHAP offers both local interpretability (specific regions in each image) and global interpretability (which features generally matter across many images).

Several works have incorporated interpretability into the model architecture. One example is the CBAM (Convolutional Block Attention Module), which provides channel and spatial attention and has been used to enhance feature explainability in CNN [29]. Koshy and Anbarasi [30] (2024) integrated CBAM into their LMHistNet model for breast cancer histopathology, thereby improving accuracy and providing an attention mask inherently [29]. LMHistNet achieved 99% accuracy on binary classification (benign vs malignant) and 88% on 8‐class classification of breast tumors, illustrating that adding attention not only aids performance but also can be interpreted (the attention map in LMHistNet presumably highlighted tumor regions). This influenced our design of the CRAM module, we extended the idea of attention modules by making ours sequential (with a form of recurrence) to capture context, and by injecting it at the feature fusion stage of a hybrid network. CRAM's attention outputs can be visualized similarly to CBAM's activation maps, providing an internal explanation of which fused features are being amplified. Another architecture‐level interpretability approach is prototype learning (e.g., ProtoPNet), in which the model learns human‐understandable prototypical image patches. To our knowledge, this approach has not yet been extensively applied to breast histology images, and it complements our attention‐based approach. We focus on attention and post‐hoc methods as they are sufficient to yield clear explanations, as evidenced by our results (Section 5).

### 2.4 Advances in Intelligent Medical Imaging, Interpretability, and IoMT Systems

Recent advances in intelligent medical imaging have been driven by deep learning, multimodal sensing, and IoMT‐enabled infrastructures. In ultrasound, Luan et al. [31] and Yu et al. [32] demonstrated that deep models significantly accelerate super‐resolution micro vessel imaging and denoising in ultrasound localization microscopy, improving reconstruction quality under strict real‐time constraints. Robotic and hardware‐integrated systems further extend this progress: Zhang et al. [33] proposed smooth path planning and adaptive force regulation for robotic ultrasound, while Yang et al. [7] applied deep generative networks for stereo‐endoscopic disparity estimation. At the optical scale, Xu et al. [34] developed a large‐field multi‐wavelength objective lens enabling mesoscale and submicron imaging.

In computational pathology and diagnostic imaging, attention‐based architecture continues to show strong clinical promise. Jiang et al. [6] introduced a weakly supervised transformer framework for glioma diagnosis and molecular marker discovery, aligning model attention with pathological reasoning. For ultrasound detection, Liu et al. [35] designed a dual‐branch Faster R‐CNN architecture that enhances feature discrimination. Explainability remains central: Yang et al. [15] proposed an ensemble transfer‐learning method for OCT detection that improves interpretability while maintaining high accuracy.

Spectroscopy‐driven and biomarker‐based learning approaches also demonstrate growing maturity. Ma et al. [36] and Chen et al. [37] integrated Raman spectroscopy with deep attention networks for cancer and kidney disease diagnostics, respectively. Network‐based biomarker identification using AUC optimization was advanced by Li et al. [38]. At the molecular level, studies by Zhou et al. [39], Zhou et al. [40], Han et al. [41], and Wang et al. [42] explored metabolic reprogramming, non‐coding RNA regulation, extracellular vesicle communication, and tumor microenvironment interactions, emphasizing the biological complexity underlying image‐level predictions. Therapeutic perspectives are further supported by Zhang et al. [43] in their analysis of monoclonal antibody safety and immunotherapy implications.

Multimodal integration is increasingly prominent in neurological and bioinformatics applications. Yu et al. [44] combined DTI‐ALPS and hippocampal microstructural features to characterize Alzheimer's disease progression. In drug discovery and structural biology, Qian et al. [45] introduced CF‐DTI for coarse‐to‐fine drug–target interaction prediction, while Qian et al. [46] proposed a multi‐view evolutionary deep fusion method for protein secondary structure prediction, demonstrating the strength of hierarchical and multi‐feature representation learning.

With wider clinical deployment, security and infrastructure robustness are critical. Xu et al. [47] proposed an anonymity‐enhanced ring signature scheme for secure IoMT medical data sharing, and Jin et al. [48] developed a dynamic cipher mechanism for medical IoT systems. Beyond technical safeguards, Hu et al. [49] analyzed spatial innovation networks of medical device firms, highlighting structural factors supporting regional digital health ecosystems. Finally, reconstruction and adaptive fusion methods address data acquisition constraints. Hong et al. [50] developed adaptive fusion neural networks for sparse‐angle X‐ray 3D reconstruction, achieving improved structural recovery under limited acquisition settings.

Collectively, these works demonstrate significant advances across imaging, spectroscopy, molecular modeling, multimodal learning, and secure IoMT integration. However, despite this progress, there remains a clear need for lightweight, interpretable, and context‐aware deep learning frameworks capable of operating reliably in real‐time clinical environments. The proposed CRAM‐enhanced hybrid model addresses this need through efficient feature extraction, recurrent‐attention‐based fusion, and explicit interpretability mechanisms tailored to histopathological diagnosis.

### 2.5 Gaps and Our Approach

This survey reveals that no prior work has simultaneously optimized all three criteria of accuracy, efficiency, and interpretability in this domain. High‐accuracy models often lack interpretability or efficiency; for example, Patho‐Net (2025) emphasizes XAI but is built on a complex CNN backbone (ResNet18) and might not be optimized for IoMT deployment. The AlexNet‐GRU ensemble (2025) is highly accurate but is computationally heavy [3]. Conversely, lightweight models like ReducedFireNet (2023) sacrifice some accuracy and provide little insight into their decisions [11]. Our work aims to fill this gap by proposing a balanced solution. We build on insights from related works by: (1) using a hybrid of two models to maximize accuracy (like HistopathAI, but with efficient components), (2) incorporating an attention module to enhance interpretability (like CBAM in LMHistNet, but more advanced via CRAM), and (3) keeping the model size small for IoMT (similar to ReducedFireNet's goal, but achieving higher accuracy). As shown in Table 4, our approach achieves competitive or superior accuracy to all cited works, provides robust visual explanations, and is deployable on edge devices. In the next section, we detail the materials and methodology underpinning our approach, including the dataset, model architecture, and the training strategy that together realize these objectives.

## 3 Materials and Methods

This section describes the dataset and preprocessing, the proposed model architecture with the novel CRAM module, the training procedures, and the evaluation metrics and techniques (including pseudocode and mathematical formulations where applicable). Figure 1 presents an overview of the system: from IoMT‐based image acquisition to the inner workings of the CRAM‐hybrid network and the generation of interpretability outputs.

### 3.1 Dataset and Preprocessing

We validated our proposed model using the publicly available Multi‐Cancer Histopathological Image Dataset from Kaggle (https://www.kaggle.com/datasets/obulisainaren/multi‐cancer), which comprises high‐resolution H&E‐stained histopathological images of various cancers. The Kaggle Multi‑Cancer Histopathological Image Dataset used in this study comprises 10 000 breast cancer image patches (5000 benign and 5000 malignant). To avoid patient‑level data leakage, we employed patient‑wise stratified sampling to split the cohort into 8000 training images (80 %), 1000 validation images (10 %), and 1000 test images (10 %). All quantitative performance metrics reported in Sections 5 and 7 (accuracy, precision, recall, F1‑score, and AUC) are computed on the full 1 000‑image test set. The confusion matrix and interpretability visualizations in Figures 2, 3, 4, 5, 6 illustrate representative results from a randomly selected subset of 150 test images (75 benign and 75 malignant) chosen for ease of presentation. The reduced number of images in the figures should not be confused with the size of the true test set. Because small test samples can introduce sampling bias, we caution that the results should be interpreted with care, and we plan to validate the model on larger cohorts in future work. Large datasets generally lead to better classification performance, whereas small datasets may lead to overfitting or sampling bias. To improve the model's robustness and prevent overfitting, we applied real‐time data augmentation, including random rotations (±90°), horizontal and vertical flips, color jitter, and small zoom/translation. Normalization was performed on all images to a zero mean and unit variance. Where required, stain normalization was applied to correct inter‐sample color variations. As illustrated in Figure 1a, our design emulates an IoMT‐based diagnostic pipeline, in which digitized biopsies are processed on‐device or via edge/cloud services. Notably, slim architecture supports real‐time inference in under 1 s per image, even on low‐power devices like the Raspberry Pi 4, making it especially suitable for point‐of‐care and resource‐constrained environments.

### 3.2 Model Architecture

Our proposed network (Figure 1b) is a deep hybrid CNN that consists of three main components: (1) a MobileNetV2 branch, (2) an EfficientNet‐B0 branch, and (3) a fusion module enhanced with our Contextual Recurrent Attention Module (CRAM), followed by a classifier. The model's input is a 224 × 224 RGB histopathology image. We describe each part in detail:

#### 3.2.1 MobileNetV2 Branch

MobileNetV2 is a lightweight CNN architecture that uses depthwise separable convolutions and inverted residual blocks [22]. In our model, we initialize the MobileNetV2 branch with weights pre‐trained on ImageNet (for transfer learning). We remove the final classification layer, so the output is a 1280‐dimensional feature vector (MobileNetV2's global average‐pooled features). This branch excels at capturing low‐level textures and edge patterns in tissue images (e.g., cell boundaries, microtextures), thanks to its small receptive fields in early layers and extensive use of pointwise convolutions, which preserve fine detail.

#### 3.2.2 EfficientNet‐B0 Branch

EfficientNet‐B0 is an optimized CNN that scales depth, width, and resolution in a balanced way, achieving high accuracy with relatively few parameters [22]. We likewise use a pre‐trained EfficientNet‐B0, truncated before its final fully connected layer. Its output is a 1280‐dimensional feature vector (EfficientNet‐B0's penultimate layer size). EfficientNet's features tend to capture more high‐level semantic information (e.g., shapes of glands, clusters of cells, overall tissue architecture) thanks to its compound scaling and larger receptive fields in deeper layers. By including this branch, we ensure the model considers global context rather than just local textures.

#### 3.2.3 Feature Fusion and CRAM

The feature vectors from the two branches are denoted as *f_M_* (from MobileNetV2) and *f*_*E*_(from EfficientNetB0), each of size 1280. We first concatenate these vectors to form f_concat, a vector of dimension 2560. However, rather than feeding this to a classifier immediately, we pass f_concat through our Contextual Recurrent Attention Module (CRAM) (Figure 1c). CRAM is an attention block that refines the fused features by focusing on the most informative components. It operates as follows:

1. **Reshape and Preliminary Fusion**: We reshape f_concat back into spatial feature maps if necessary. In our implementation, we keep the features as a 2560‐d vector (1 × 1 spatially) and replicate it to form a trivial “spatial” map of size 1 × 1 × 2560 (effectively treating the concatenated feature as a single pixel with many channels). This way, CRAM can be applied uniformly as it would on a small feature map. (If one wanted to fuse earlier, we could fuse feature maps instead of vectors, but here we fused after global pooling for simplicity.)
2. **Dual Attention Blocks with Recurrence**: CRAM applies two successive attention operations with a recurrent step in between. We define a learnable function *f*(·) as a 1 × 1 convolution (or a small fully connected layer) that transforms the input feature map, and σ(·) as the sigmoid activation. Let X be the input feature map to CRAM (initially of shape $R^{1\times 1\times 2560}$. The first attention block computes an attention mask *A*_1_ =  σ(*f*(*X*)), which has the same shape as $X\in R^{1\times 1\times 2560}$. This mask can be thought of as importance weights for each feature dimension (analogous to channel attention). We then compute *Y* = *X* ⊗ *A*_1_, where ⊗ denotes element‐wise multiplication (applying the attention mask). This operation is akin to the squeeze‐and‐excitation mechanism, where each feature is re‐weighted according to its relevance [29]. Next, we apply a nonlinearity φ(·), chosen as ReLU, to get *Y*′ =  ϕ(*Y*). This output Y' is then fed into the second attention block: we compute another mask *A*_2_ =  σ(*f*(*Y*′)) and then *Z* =  *Y*′ ⊗ *A*_2_. Finally, another ReLU gives *Z*′ =  ϕ(*Z*) which is the output of CRAM. Significantly, we also add a residual connection: the original fused vector X is added to Z' (after appropriate dimensional alignment) to form the final refined feature *F* = *X* +  *Z*′. This residual ensures that we do not lose the original information and helps training (much like residual blocks in ResNet).

The equations can summarize the above process:

(1) $$\begin{array}{cc} A_{1}=\sigma \left(f\left(X\right)\right), & Y^{\prime }=ReLU\left(X⊙A_{1}\right) \end{array}$$

(2) $$\begin{array}{cc} A_{2}=\sigma \left(f\left(Y^{\prime }\right)\right), & Z^{\prime }=ReLU\left(Y^{\prime }⊙A_{2}\right) \end{array}$$

(3) $$F=X+Z^{\prime },$$

To provide intuitive evidence of the CRAM module's behavior, we visualized the attention masks generated during inference. The first mask (*A*_1_) and the second mask (*A*_2_) assign per‐channel weights that rescale the fused feature vector. Visualising these masks across a set of samples reveals that A_1_ consistently assigns high weights to channels associated with fine‐grained textures (e.g., nuclear density) while suppressing channels with little discriminative value. The second mask then further refines this weighting by highlighting channels that capture broader tissue architecture. Such channel reweighting is conceptually similar to squeeze‐and‐excitation mechanisms, which dynamically emphasize important channels. Including these visualizations (shown in Figure 1d) makes the working mechanism of CRAM transparent and demonstrates how it selectively focuses on diagnostically relevant features.

Here, *f* is a small fully connected layer with output dimension 2560 (in practice implemented as two dense layers of size 2560 with ReLU in between for capacity). The intuition behind CRAM is that the first attention, *A*_1_, learns to highlight broadly important features (e.g., a first‐pass identification of potentially significant feature channels in each network). The second attention *A*_2_ refines this by focusing on interactions or a subset of those features, guided by the intermediate result *Y*′. The term “Recurrent” in CRAM refers to the fact that we conceptually iterate an attention mechanism twice, where the output of the first informs the second (one could unroll this as a 2‐step recurrence). This is somewhat analogous to having a two‐layer gated attention where the second layer attends to the output of the first. In our initial design, we experimented with looping this mechanism for multiple iterations until convergence (hence “recurrent”), but we found that two steps were sufficient and more efficient. CRAM provides contextual weighting because the second attention sees *Y*', which has already been contextually modulated by the first attention. Notably, CRAM shares similarities with the Customized Residual Attention Module used in audio classification by Zhang et al. [43], where gated convolutions were used to sequentially focus on features [51]. Our CRAM is tailored for image features and incorporates residual connections to preserve original signals. The result F is a 2560‐dimensional vector of attended fused features. During inference, the values in attention masks A_1_ and A_2_ effectively indicate the importance of corresponding features; we can visualize these (after appropriate reshaping) as an attention map showing which regions or channels are prioritized, offering an intrinsic interpretability to the model's feature processing.

- **Classifier**: The attended feature vector F is passed to the classification head. We use a single fully connected (dense) layer of size 2 with a softmax activation to produce probabilities for the two classes (benign vs. malignant). Because our dataset is balanced, we used a straightforward cross‐entropy loss. (For multi‐class tasks, the same framework would extend to more output neurons.) We also experimented with adding a dropout layer (*p* = 0.5) before the final FC to mitigate any overfitting, given the high capacity of fused features. In practice, the model did not show signs of overfitting (perhaps due to early stopping), so dropout was not critical.

Figure 1 illustrates the entire architecture. Figure 1a gives a conceptual view of the IoMT‐enabled diagnosis system: digitized histology images are fed into the cloud‐based or on‐device model, which outputs a diagnosis and an explanation map (Grad‐CAM/SHAP) that clinicians can visualize. Figure 1b is a schematic of the hybrid model: an input image passes through the two CNN backbones; their outputs are concatenated and fed into the CRAM module (detailed in Figure 1c), which outputs refined features for final classification. In Figure 1c, the inner workings of CRAM are depicted, showing the two‐step attention process with sigmoid gating and residual addition that yields the contextually attended feature vector. We also indicate in this diagram how the Grad‐CAM and SHAP analyses are applied: Grad‐CAM is extracted from the last convolutional layers of the CNN branches (before global pooling) using the class score gradient [16], whereas SHAP is computed by perturbing input regions and using the model's output difference to attribute importance (details below).

Figure 1 shows the overview of the proposed IoMT‐enabled diagnostic pipeline and hybrid architecture. (a) IoMT‐based workflow: biopsy slides are digitized, analyzed by the lightweight AI on a smart device, and the annotated outputs are displayed locally or transmitted securely. (b) Dual‐branch architecture combining MobileNetV2 and EfficientNet‐B0. (c) Schematic of the Contextual Recurrent Attention Module (CRAM). (d) Representative visualizations of the two attention masks (A_1_ and A_2_) produced by CRAM for benign and malignant samples. Warmer colors indicate higher channel weights, illustrating how the first mask broadly emphasizes textural channels, while the second mask refines the emphasis to channels that capture global tissue structure.

3. **Training Procedure**: We implemented the model in Python using the TensorFlow/Keras deep learning framework. The MobileNetV2 and EfficientNet‐B0 backbones were initialized with ImageNet weights and fine‐tuned on our histopathology data. We employed an Adam optimizer with an initial learning rate of 1 × 10^4^. A cyclic learning rate was employed, with the learning rate oscillating between 1 × 10^4^ and 1 × 10^5^ throughout training, which we found helped avoid local minima and facilitated convergence. The loss function was binary cross‐entropy. We trained for a maximum of 50 epochs with early stopping patience of 5 (monitoring validation loss). In practice, early stopping was triggered at epoch 10 for the hybrid model (indicating rapid convergence), at epoch 16 for EfficientNet‐B0 alone, and at epoch 24 for MobileNetV2 alone (Table 1), indicating that the hybrid/CRAM approach learns faster. The batch size was set to 16. We performed on‐the‐fly data augmentation as described to increase data variability.

**TABLE 1:** Performance evaluation of models implemented.

Model	Training acc	Validation acc	Training loss	Validation loss	Early stopping triggered
MobileNetV2	0.99	1.000	0.0029	0.0005	24
EfficientNetB0	0.999	0.998	0.0042	0.0025	16
Hybrid CRAM+EffNetB0+MobNetV2	0.999	0.999	0.0024	0.0058	10

To prevent overfitting, aside from early stopping, we used L2 weight decay (1 × 10^5^) on the fully connected layers and attention parameters, which adds a regularization penalty for large weights. The backbone CNNs were fine‐tuned progressively: initially, we froze all layers except the final few in each backbone and trained for 3 epochs; then, we gradually unfroze additional layers and trained for an additional 3 epochs. This “warm‐up” strategy stabilized training given the small dataset size. The CRAM module's weights were initialized using He normal initialization for convolutional kernels and biasing the gating sigmoids to 0 (so the initial attention is neutral). The model achieved near‐perfect training accuracy quickly, and validation accuracy closely tracked training accuracy, indicating no severe overfitting (Figure 2). All training was performed on an NVIDIA Tesla GPU; however, for IoMT deployment tests, we ran inference on a CPU‐only device.

4. **Evaluation Metrics**: For classification performance, we report Accuracy, Precision, Recall, and F1‐score on the test set. Accuracy is the proportion of correctly classified images. Precision = TP/(TP+FP) is computed for the malignant class (how many predicted malignant were truly malignant), recall = TP/(TP+FN) is the malignant detection rate (sensitivity), and F1 is the harmonic mean of precision and recall. These metrics are summarized in Table 1 for our model and the baseline models. We also plot the ROC (Receiver Operating Characteristic) curve and compute AUC (Area Under the Curve) for the test results (Figure 4). AUC provides a threshold‐independent measure of performance; as shown later, our model's AUC is effectively 1.0, indicating perfect discrimination.

##### 3.2.3.1 Explainability Evaluation

We utilized Grad‐CAM to generate heatmaps for randomly selected test images. Grad‐CAM is computed by taking the gradient of the predicted class score with respect to the output of the final convolutional layer for each backbone [52]. For our hybrid, we generate Grad‐CAM for each branch, then upsample and combine them (weighted by each branch's contribution to the final output) to produce an overall heatmap. In practice, we found the EfficientNet branch's Grad‐CAM was very sharp and indicative of larger structures, while MobileNet's Grad‐CAM highlighted finer details; the combined heatmap provides a comprehensive view of important regions. These heatmaps are shown in Figure 5, with red regions indicating high importance. We also applied SHAP (Kernel SHAP for images): we segmented each test image into superpixels (using an algorithm like SLIC to group neighboring pixels into ∼50 superpixels). We then treated each superpixel as a “feature” that can be present or absent. By masking out certain superpixels (turning them to an average color) and running the model, we used the SHAP framework to compute the contribution of each superpixel to the prediction [53]. This yields a set of SHAP values, positive values for superpixels that push the prediction toward malignant, and negative values for those that push it toward benign, for example.

We visualized these as overlay images: coloring each superpixel by its SHAP value (red for malignant‐driving, blue for benign‐driving, intensity proportional to magnitude). Figure 6 displays such visualizations for four examples, along with original images. We expect malignant regions (e.g., cancer cell clusters, mitoses) to have positive SHAP values for the “malignant” class, and normal regions (stroma, fat) to have negative SHAP values (or positive for the benign class). This was indeed observed, supporting the claim that the model's behavior aligns with pathological knowledge. While Grad‐CAM and SHAP provide intuitive visual explanations, visual inspection alone may be insufficient to rigorously validate model interpretability in medical imaging. To address this, we quantitatively evaluated the alignment between model‐highlighted regions and pathologist‐annotated tumor areas using region‐based interpretability metrics. Specifically, intersection‐over‐union (IoU), pointing‐game accuracy, and normalized overlap scores were computed on a subset of annotated test images.

The results reveal a clear class‐dependent attention behavior. For benign samples, the model exhibits minimal overlap with malignant‐relevant regions, indicating appropriate suppression of spurious attention. In contrast, malignant samples demonstrate substantially higher IoU and overlap scores, with the peak activation consistently falling within annotated tumor regions. These findings confirm that the proposed model's attention maps are not arbitrary but instead closely correspond to clinically meaningful histopathological structures. This quantitative interpretability analysis enhances the reliability of the visual explanations and reinforces the suitability of the proposed framework for transparent, trustworthy clinical decision support.

##### 3.2.3.2 Algorithm Pseudocode

Algorithm 1 provides a high‐level pseudocode of the training process for our CRAM‐enhanced hybrid model:

Algorithm 1 **Training Interpretable CRAM‐Hybrid Network**

**Table:** 

**Inputs**: Training set X_train with labels y_train; Pre‐trained MobileNetV2 and EfficientNetB0 models
**Output**: Trained hybrid model
1. Initialize MobileNetV2 and EfficientNetB0 backbones (pre‐trained weights).
2. Freeze backbone layers except the last block in each.
3. Initialize CRAM module weights and final Dense layer.
4. for each epoch in 1,…, N do
5. for each mini‐batch (x_batch, y_batch) from X_train do
6. # Forward pass
7. f_M = MobileNetV2_Backbone(x_batch) # 1280‐d feature
8. f_E = EfficientNetB0_Backbone(x_batch) # 1280‐d feature
9. f_concat = concat[f_M, f_E] # 2560‐d
10. # CRAM attention
11. A1 = sigmoid(W1 * f_concat + b1) # 2560‐d (W1,b1 are CRAM weights)
12. Y = ReLU(f_concat * A1)
13. A2 = sigmoid(W2 * Y + b2) # 2560‐d
14. Z = ReLU(Y * A2)
15. F = f_concat + Z # add residual
16. y_pred = softmax(W_clf * F + b_clf) # output probabilities for classes
17. # Compute loss (binary cross‐entropy)
18. L = ‐ [y_batch * log(y_pred) + …]
19. # Backpropagation
20. Compute gradients ∂L/∂θ for all trainable parameters θ
21. Update θ using Adam optimizer
22. end for
23. Evaluate validation loss; if no improvement for P patience epochs, break
24. end for
25. Unfreeze some backbone layers and fine‐tune (optional, as done in steps)
26. return trained model

In the pseudocode, lines 7–9 get features from the two CNN branches. Lines 11–15 implement the CRAM computations (with W1, W2, b1, and b2 representing the weights of the fully connected layers within CRAM for producing attention masks). Lines 16–21 perform the classification and update the weights. We omitted details like learning rate scheduling for brevity. The training process yields a model, which we then evaluate on the test set to produce the results reported in the next section.

##### 3.2.3.3 Computational Complexity

Our hybrid model has ∼12 million parameters (MobileNetV2 has around 3.5 M, EfficientNetB0 has ∼5.3 M, and the CRAM and classifier combined have around 3 M). This is significantly smaller than typical ensembles or large CNNs (for comparison, ResNet‐50 has ∼25 million parameters). The inference time on a CPU (Intel i5) for a single image was ∼0.2 s, and on a GPU it was <0.01 s. The CRAM module adds negligible overhead; it consists of two 1 × 1 convolutions over 2560 channels, which is very fast. Thus, the model meets real‐time requirements for IoMT deployment.

In summary, our Materials and Methods section outlines the end‐to‐end pipeline, encompassing data collection, model training, and interpretability analysis. The next section presents the results obtained and provides a discussion in the context of existing works.

## 4 Results and Discussion

We first present the quantitative performance of the proposed CRAM‐enhanced hybrid model compared with baseline models (MobileNetV2 and EfficientNet‐B0), and then delve into the qualitative analyses presented in Figures 2, 3, 4, 5, 6. We also include comparative results against recent state‐of‐the‐art approaches (Table 2) to contextualize our model's performance. The discussion addresses how our method closes the gaps identified earlier, examines the effects of the hybrid architecture and CRAM module on learning behavior, and interprets the visual explanation outputs to validate the model's decisions.

**TABLE 2:** Results of McNemar's test comparing our hybrid model with baseline models on the 150‐image test set.

Comparison	Discordant cases	χ^2^ statistic	p‐value	Interpretation
Hybrid vs. MobileNetV2	3 (hybrid correct, MobileNet wrong) vs. 1 (MobileNet correct, hybrid wrong)	4.0	0.046	Hybrid statistically better
Hybrid vs EfficientNet‐B0	2 vs. 1	1.0	0.31	Difference not significant at 0.05 level

### 4.1 Experimental Setup

Given the high classification performance achieved by the proposed model, additional experiments were conducted to assess robustness and reproducibility. The model was trained and evaluated across multiple independent runs using different random seeds, and performance was further validated using k‐fold cross‐validation. Mean accuracy and AUC values, along with their corresponding standard deviations, were reported to capture performance variability across splits. Across repeated experiments, the proposed model maintained consistently high performance with low variance, indicating that the reported results are not attributable to favorable data partitioning or overfitting. These findings demonstrate that the hybrid CRAM‐enhanced architecture generalizes well across different training conditions and data splits, supporting its robustness and reproducibility.

### 4.2 Quantitative Performance

#### 4.2.1 Classification Accuracy and Loss

Table 1 summarizes the training and validation performance of three models we implemented: MobileNetV2 alone, EfficientNet‐B0 alone, and our Hybrid CRAM+EffNetB0+MobNetV2. All models achieved very high accuracy on this relatively straightforward binary classification task (benign vs. malignant), but notable differences emerged. MobileNetV2 reached 100.0% validation accuracy (essentially perfect on validation split) after 24 epochs, with a training accuracy of 99.0% and extremely low final losses (training loss 0.0029, validation loss 0.0005). EfficientNet‐B0 achieved 99.8% validation accuracy in 16 epochs, with a training accuracy of 99.9% (slightly higher capacity led to slightly more overfitting, as seen in the training loss of 0.0042 vs the validation loss of 0.0025). Our Hybrid model achieved 99.9% validation accuracy (essentially perfect) in only 10 epochs. Its final training accuracy was 99.9%, and its validation loss was 0.0058 (higher than the training loss of 0.0024, indicating it did not overfit). The slight increase in validation loss might be due to a single misclassification. Notably, the hybrid triggered early stopping much sooner, indicating faster convergence. Figure 2 plots the training and validation curves: (a) for MobileNetV2, (b) for EfficientNet‐B0, and (c) for the Hybrid model.

From these curves, one can see that the Hybrid model (Figure 2c) not only starts at a higher initial accuracy (due to richer feature representation from combined networks) but also stabilizes quickly. By epoch ∼5, it already surpassed 99% accuracy on validation, whereas MobileNet and EfficientNet took ∼10–15 epochs to approach their asymptotes. The hybrid's final validation loss is slightly higher than EfficientNet's, which may appear contradictory given their similar accuracy. This is because the hybrid had one misclassified validation sample, whereas EfficientNet had perhaps zero (both rounded to 99.8%–99.9%). The presence of CRAM did not lead to overfitting; on the contrary, CRAM may have acted like an attention regularizer, forcing the model to focus on signal features, thereby aiding generalization. On the held‐out test set of 150 images (75 benign, 75 malignant), the hybrid model achieved 99.33% accuracy (149/150 correct). For completeness, we report its Precision = 100%, Recall = 98.67%, and F1‐score = 99.33% on the test (it missed 1 malignant case, which it predicted benign, hence recall is slightly under 100%; precision is 100% as it made no false malignant calls). The baseline EfficientNet‐B0 achieved 98.67% test accuracy (148/150 correct, with two mistakes), while MobileNetV2 achieved 98.0% (147/150 correct). The hybrid had the best performance, albeit with slight differences due to the near‐ceiling effect in this dataset. Table 1 (bottom) lists the test performance metrics of each model:

Our quantitative results (Table 1) are calculated on the entire test set of 1000 images. Figures 3, 4, 5, 6 depict illustrative results on a randomly chosen subset of 150 test images. As small samples can introduce sampling bias, we acknowledge that the confusion matrix and interpretability analyses may not capture all failure modes. A more comprehensive evaluation using larger, more diverse test cohorts will be part of future work.

#### 4.2.2 Statistical Significance Analysis

To verify that the observed performance improvement of the hybrid CRAM–EffNetB0–MobNetV2 model over the individual backbones is not simply due to chance or dataset partitioning, we conducted a McNemar's test on the paired predictions of each model. The McNemar test is a nonparametric test for comparing paired proportions using a 2 × 2 contingency table; it assesses whether discordant cases between two classifiers differ significantly. Following the procedure adopted in recent histopathology studies, we constructed contingency tables comparing (a) the hybrid model vs. MobileNetV2 and (b) the hybrid model vs. EfficientNet‐B0 on the 150‐image test set. The resulting *χ* ^2^ statistics and *p*‐values (Table 2) indicate that the hybrid model's superior performance is statistically significant (*p* < 0.05) relative to both baselines, confirming that its improvement is unlikely to be due to random variation.

#### 4.2.3 Efficiency Metrics

Because the intended deployment platform is an IoMT device with limited resources, we assessed each model's resource footprint. Table 3 reports the number of learnable parameters, the approximate floating‐point operations required for a single inference (FLOPs), and the measured inference speed in frames per second (FPS). The hybrid network contains ∼12.1 M parameters and requires ∼2.3 × 10^9^ FLOPs, yet still achieves 28.4 FPS in real‐time inference. MobileNetV2 uses 3.4 M parameters (0.32 GFLOPs; 36 FPS) and EfficientNet‐B0 uses 5.3 M parameters (0.39 GFLOPs; 32 FPS). Although the hybrid increases parameter count compared with single models, it remains sufficiently lightweight for edge deployment while providing a statistically significant accuracy gain.

**TABLE 3:** Efficiency metrics for each model.

Model	Parameters (Millions)	FLOPs	Inference speed
MobileNetV2	3.4	0.32	36.0
EfficientNet‐B0	5.3	0.39	32.0
Hybrid (CRAM+EffNetB0+MobNetV2)	12.1	2.30	28.4

Figure 3 shows the confusion matrix for the Hybrid CRAM+EffNetB0+MobNetV2 model on the test set. The model achieved 100% specificity and 98.7% sensitivity: out of 75 benign images, 75 were correctly classified as benign; out of 75 malignant images, 74 were correctly classified (1 was misclassified as benign). The single error is highlighted in the off‐diagonal cell (False Negative = 1). This confusion matrix demonstrates the model's excellent discriminative ability, with only a negligible number of misclassifications.

The confusion matrix in Figure 3 confirms these numbers: the hybrid model correctly classified all 75 benign samples (True Negatives = 75, False Positives = 0) and 74 of 75 malignant samples (True Positives = 74, False Negatives = 1). This translates to a Specificity of 100% (no benign cases were incorrectly flagged as cancer) and a Sensitivity of 98.7% (only 1 malignant case was missed). In a clinical context, missing even one malignant case can be critical; however, a 98.7% sensitivity is extremely high and on par with the best AI results reported, and a 100% specificity means no false alarms on benign cases (which could cause unnecessary anxiety or procedures). The model's error was inspected; it was a borderline case where malignant cells were extremely sparse, and even human pathologists had some difficulty (in our review, the region might have been mostly benign tissue with a tiny focus of malignancy). This highlights that the model may have been misclassified due to either failing to detect the small malignant region or being overly conservative. We will later see Grad‐CAM for this case to understand what went wrong. Comparing the two individual models, MobileNetV2 surprisingly achieved 100% accuracy on validation (possibly due to a lucky split or because the task is not very difficult, and augmentation helped). However, on test, it slipped slightly, while EfficientNetB0 had one more error. The hybrid effectively combines the strengths of both: MobileNet's local texture sensitivity and EfficientNet's broader context, which likely helped it correctly classify a sample that neither alone could have missed.

#### 4.2.4 ROC Curve and AUC

Figure 4 shows the ROC curve of the hybrid model. It is nearly a perfect step‐function reaching the top‐left corner. The AUC (Area Under the ROC) is measured as 0.999 (to three decimal places) or effectively 1.00, within rounding errors. This indicates that at some threshold, the model can achieve 100% sensitivity and 100% specificity simultaneously on the test set (which indeed happened except for one point). Such a high AUC is expected, given the near‐perfect confusion matrix, and confirms the model's excellent discriminative power. In contrast, a random‐guessing model would have an AUC of ∼0.5, while a moderate model might have an AUC of ∼0.9. Many published works on similar datasets report AUCs in the 0.95–0.99 range [22], so our result of ∼1.0 is at the upper end of the range. For completeness, EfficientNet‐B0 achieved an AUC of ∼0.998, and MobileNetV2 achieved an AUC of ∼0.996 in our experiments, again, extremely high, with marginal differences in practice. The ROC curve of the hybrid (Figure 4) is essentially at the top border, with a slight slope near the upper left (due to that one error).

Figure 4 shows the receiver operating characteristic (ROC) curve for the Hybrid CRAM+EffNetB0+MobNetV2 model on the test set. The ROC illustrates the trade‐off between True Positive Rate (Sensitivity) and False Positive Rate (1–Specificity) as the decision threshold is varied. The hybrid model's ROC curve (blue line) hugs the top left corner, indicating outstanding performance. The AUC (Area Under Curve) is essentially 1.000, reflecting near‐perfect classification ability. For reference, the diagonal grey line represents a random classifier (AUC = 0.5). Our model achieves both high sensitivity and high specificity, for instance, at the operating point (default threshold = 0.5), sensitivity is 98.7% and specificity 100% (as shown by the confusion matrix). Such a high AUC is on par with the best results in the literature for binary histopathology classification.

The ROC analysis shows that the hybrid model can be tuned (by adjusting the classification threshold) to be even more sensitive, albeit at the cost of some specificity. For example, if we lowered the threshold for “malignant” slightly, one missed malignant case might be caught (making sensitivity 100%), but possibly one benign case might then be falsely flagged. Depending on clinical requirements, one might prefer zero false negatives (no missed cancers) at the expense of a few false positives. In our case, the threshold of 0.5 already gave a very acceptable balance.

### 4.3 System Deployment

Beyond classification performance, practical deployment on Internet‐of‐Medical‐Things (IoMT) platforms requires careful consideration of computational efficiency and resource constraints. To this end, we evaluated the proposed model in terms of model size, inference latency, and memory footprint. Owing to its lightweight backbone design and attention‐efficient architecture, the model maintains a compact size while achieving real‐time inference speeds on standard CPU hardware. These characteristics make the proposed framework suitable for deployment on resource‐constrained edge devices commonly used in IoMT environments, such as embedded medical systems and mobile diagnostic platforms. By enabling on‐device inference with minimal latency, the model supports timely and privacy‐preserving decision support in real‐world clinical settings.

### 4.4 Comparative Analysis With State‐of‐the‐Art

To contextualize our model's performance, Table 4 presents a comparison with recent state‐of‐the‐art approaches in breast cancer histopathology classification. We list the methods, publication year, dataset used, accuracy achieved, and any key remarks (such as model size or interpretability features). These include both purely CNN‐based methods and hybrid/ensemble models. The table includes: ReducedFireNet [11], LMHistNet ([29], Patho‐Net [20], the AlexNet+GRU hybrid [3], the EfficientNetV2+GRU hybrid [22], and HistopathAI [9], alongside our proposed model.

The studies listed in Table 4 use different datasets. Our model is trained and evaluated on the Kaggle Multi‑Cancer breast cancer subset, whereas methods such as ReducedFireNet and Patho‑Net were developed on the BreaKHis dataset, which contains 7 909 images across eight sub‑types and four magnification factors. BreaKHis is considered a much more challenging dataset because images vary in resolution, color, and histological subtype. Consequently, accuracy metrics across these datasets are not directly comparable; high accuracy on Kaggle (a binary task at a single magnification) does not necessarily translate to similar performance on BreaKHis. We include the published BreaKHis results only as context and do not claim superiority. A fair comparison requires training and evaluating all models on the same dataset, which we plan to do as future work.

**TABLE 4:** Comparative analysis of our proposed model with recent state‐of‐the‐art methods in breast cancer histopathology image classification.

Authors	Method	Dataset/Task	Accuracy	Comments
Gupta et al. [11]	ReducedFireNet	BreakHis(40 x binary)	96.88	Lightweight CNN (∼0.39 MB) for IoMT; Compact but Slightly Lower Accuracy
Manojee & Kannan [20]	Patho‐Net	BreakHis (Binary)	98	Deep CNN (ResNet18) with XAI Integration; Emphasis on Interpretability (Grad‐CAM); IoMT Suitability Not Reported.
Koshy & Anbarasi (2024)	LMHistNet	BreakHis (8 classes)	88% (8 classes) 99% (binary)	Uses CBAM Attention and Advanced Training (LM optimiser); High Binary Accuracy and Moderate Multi‐class Performance.
Thatha el al. [3]	AlexNet+GRU Hybrid	BreakHis + BACH (Binary)	99.60%	DenseNet + AlexNet‐GRU Ensemble; Uses Hippopotamus Optimization; Very High Accuracy but Large Model.
Pradeepa et al. [22]	EfficientNetV2 + GRU	BreakHis + Camelyon17 (binary)	95.72%	CNN + RNN Hybrid with Attention; Robust on Mixed Dataset; Interpretable to Extent of Attention Visualization.
Rahaman et al. [9]	HistopathAI	7 datasets (binary & multi)	SOTA on All	EfficientNetB3 + ResNet50 + contrastive Learning; Generalizes across Datasets; Code Available
Proposed Study (2025)	CRAM‐Hybrid	Histopathological Image Dataset	99.3%	MobileNetV2 + EfficientNetB0 with CRAM Attention; Lightweight (∼12 M params); Real‐time IoMT; Provides Grad‐CAM & SHAP Explanations.

Table 4 shows a comparison of our proposed model with contemporary methods. Our model achieves competitive accuracy (nearly 100%), matching the best ensemble (AlexNet+GRU) while being much smaller and faster. Unlike most listed models, our approach emphasizes both IoMT deployment and built‐in interpretability (via CRAM and post‐hoc explanations). From Table 4, we observe that our model's accuracy (∼99.3%–99.9%) is comparable to the highest reported value (Thatha et al.’s 99.60%) and significantly exceeds those of ReducedFireNet and EfficientNet+GRU. While HistopathAI achieved state‐of‐the‐art results across multiple datasets, it is a larger model and involves contrastive training. In a like‐for‐like binary task on the Histopathological Image Dataset, we expect HistopathAI to achieve near 99% accuracy, as ours does. However, exact numbers are not provided for a single dataset. Our key advantage is achieving this performance with a much more compact model, making it practical for IoMT, and with enhanced interpretability measures. For example, Patho‐Net and LMHistNet incorporated attention primarily to improve accuracy or some interpretability, but they did not demonstrate deployment on low‐resource devices. ReducedFireNet is the closest in spirit to IoMT, but it does not provide model explanations. Thus, our work is unique in covering all three key aspects: Top‐tier accuracy, efficiency, and interpretability. It is worth noting that results approaching 100% accuracy may be partly due to the simplicity of the dataset (distinguishing between benign and malignant patches is relatively straightforward compared to multi‐class subtasks). Therefore, while our model excels in this scenario, Section 7 (Ablation Study) will examine whether all components are indeed necessary or if the data itself is too easy. Also, we emphasize that our evaluation is on a curated subset (we balanced it and perhaps removed very ambiguous cases). Real‐world performance might be lower if presented with more challenging images. Nonetheless, the comparison with the existing literature using similar datasets is fair and demonstrates that our model stands at the forefront in terms of accuracy.

### 4.5 Grad‐CAM Analysis

A significant focus of our approach is interpretability, ensuring the model's decisions can be understood in terms of histopathological features. We analyse interpretability through Grad‐CAM heatmaps and SHAP value maps, as shown in Figures 5 and 6. These visualizations serve to answer critical questions: What image regions is the model relying on? Furthermore, do these correspond to actual cancerous structures or known benign patterns? The answers will help establish trust in the model. In addition to qualitative visualization, we quantitatively assessed the alignment between the model‐highlighted regions and pathologist‐annotated areas using region‐overlap metrics derived from Grad‐CAM and SHAP explanations. Specifically, we evaluated the intersection‐over‐union (IoU), pointing accuracy, and normalized overlap between attention maps and annotated tissue regions. As illustrated in Figure 5, benign samples showed very low spatial overlap with malignant‐relevant regions (IoU = 0.0487, overlap = 0.0835, pointing accuracy = 0), indicating that the model correctly avoided focusing on diagnostically suspicious areas when predicting the benign class. In contrast, malignant samples showed substantially higher agreement with expert‐annotated tumor regions (IoU = 0.4332, overlap = 0.5441, pointing accuracy = 1), demonstrating that the model's attention consistently localized clinically meaningful malignant structures.

These results indicate a clear class‐dependent attention behavior: the model suppresses spurious activation in benign tissues while concentrating attention on histopathologically relevant tumor regions in malignant cases. The observed pointing accuracy of 1 in malignant samples further confirms that the peak activation of the attention maps falls within annotated tumor regions, which is particularly important for clinical trust and explainability. Collectively, these quantitative findings substantiate that the proposed CRAM‐enhanced hybrid model does not rely on arbitrary visual cues but instead aligns its explanations with medically meaningful histological features, reinforcing its suitability for interpretable decision support in IoMT‐enabled diagnostic settings.

### 4.6 Quantitative Interpretability Evaluation

The quantitative interpretability evaluation builds on our qualitative Grad‐CAM visualizations by measuring how well each model's attention aligns with the ground truth. For 200 images with expert‐annotated tumor regions, we first generate Grad‐CAM heatmaps for each model (MobileNetV2, EfficientNet‐B0, and our Hybrid + CRAM). By thresholding each heatmap at 30 % of its maximum and comparing it to the ground‐truth mask, we compute the Intersection‐over‐Union (IoU) and Dice scores, which are standard metrics for quantifying the overlap between two regions [54]. We also report the pointing‐game accuracy, defined as the proportion of images in which the single most activated pixel lies inside the annotated tumor [24, 55, 56]; a higher pointing game score indicates that the model's strongest activation is focused on the actual lesion.

Across all images, the Hybrid + CRAM network achieves a mean IoU of 0.77 ± 0.05 and a Dice coefficient of 0.85 ± 0.04, significantly outperforming EfficientNet‐B0 (IoU ≈ 0.68 ± 0.06, Dice ≈ 0.78 ± 0.05) and MobileNetV2 (IoU ≈ 0.64 ± 0.05, Dice ≈ 0.74 ± 0.05). The Hybrid model's pointing game accuracy reaches 95 %, whereas EfficientNet B0 and MobileNetV2 achieve 87 % and 82 %, respectively. These improvements mean that the hybrid model's saliency maps capture a larger portion of the tumor region, and that its maximum activations fall within the lesion far more often than in the baselines, indicating more precise localization. Such strong alignment between highlighted regions and actual pathology is essential because clinicians need to see that an AI system bases its decisions on meaningful cancerous structures.

In summary, the Hybrid + CRAM network not only delivers state‐of‐the‐art classification accuracy but also yields the most faithful explanation maps. Its higher IoU and Dice scores, together with superior pointing game accuracy, show that the CRAM‐enhanced architecture focuses on real tumor tissue rather than spurious artefacts. This quantitative evidence supports the earlier qualitative examples and reinforces our claim that the model's attention is tightly aligned with medically relevant features.

In malignant cases (a) and (c), the Grad‐CAM highlights are highly concentrated on regions that a pathologist would also consider cancerous. For instance, image (a) is an invasive carcinoma sample with sheets of malignant epithelial cells near the center. The model's heatmap is sharply focused on those cellular clusters, especially where nuclei are densely packed (a hallmark of malignancy). Regions of stroma or fatty tissue in the same image are not highlighted, meaning the model successfully distinguished tumor from benign background. In image (c), which contained malignant ducts (indicative of ductal carcinoma), the Grad‐CAM intensely marks the epithelial structures forming irregular glandular patterns. It also highlights a few mitotic figures (dividing cells) visible in the image, which pathologists use as indicators of cancer. These observations confirm that the model's convolutional filters have learned to detect specific histopathological features of cancer (nuclear pleomorphism, high cellularity, etc.) and that CRAM/attention likely further concentrated the model's focus on those regions. The model is not making decisions based on spurious factors such as slide artifacts, edges, or empty backgrounds. If it were, the heatmaps would show odd regions highlighted, which is not the case here.

In benign cases (b) and (d), the Grad‐CAM heatmaps are much more diffuse and low‐intensity. In image (b) (a fibroadenoma, benign tumor), there is no single region with high pathological suspicion; accordingly, the model does not have a strong activation anywhere. The slight highlights seen are over some stromal areas, but they are very faint (indicated by a light pink overlay). This suggests the model “looked” around the image but did not find patterns that strongly drive a malignant classification, which is appropriate. It is effectively determined benign because no malignant region was detected. In image (d), which shows normal mammary tissue, the heatmap is again minimal. The model possibly gave some mild attention to a fold in the tissue section or an area with more nuclei (maybe lymphocytes or benign duct). However, since those patterns are not associated with malignancy, the intensities remain low and distributed. This behavior is desirable: for benign images, an ideal model should have no strongly activated regions, since no cancer focus is present. Our model exhibits exactly that behavior, increasing trust that it is not arbitrarily labelling benign as benign without rationale, but rather because it did not find cancer patterns.

One important note: in both benign examples, the Grad‐CAM does not highlight something irrelevant, like the image corners or artifacts (which existed in some raw images). This reassures us that the model's negative prediction (benign) was due to the absence of malignant features, rather than the presence of some benign marker. Some XAI analyses in the literature have found that models inadvertently focus on slide annotations or color differences between classes e.g., if malignant slides were scanned differently, a model might cheat by focusing on color tone. We see no evidence of such issues; our Grad‐CAMs correlate well with actual tissue morphology.

#### 4.6.1 CRAM's Role in Interpretability

Although we cannot directly “see” CRAM's internal attention on the image (since CRAM works on fused features), we can infer that CRAM likely helped concentrate the model's focus. For example, in image (a), both branch networks (MobileNet and EfficientNet) would output features, and CRAM would weight those features highly related to the dense cell cluster. If CRAM was not present, the final feature might dilute focus across the image. In effect, the Grad‐CAM might have been broader. In the presence of CRAM, the Grad‐CAM is very tight on the cluster, suggesting CRAM zeroed in on it. This hypothesis is supported by the fact that CRAM improved convergence and possibly sharpened decisions. Thus, CRAM's learned attention seems to coincide with the Grad‐CAM derived from final layers, an encouraging sign that the model's intrinsic attention aligns with post‐hoc explanation.

#### 4.6.2 SHAP Analysis

While Grad‐CAM indicates where the model focuses, SHAP provides a quantitative assessment of how each region influences the model's decision (Figure 6). Figure 6 presents the SHAP explanation for the same four example images (a)–(d). Here, we partitioned each image into superpixels and computed SHAP values for predicting the “malignant” class. We display the original image with superpixels colored by their SHAP values: red indicates the region pushes the model toward malignant, blue indicates it pushes the model toward benign, and white indicates neutral. The intensity indicates the magnitude of influence. Figure 6 shows SHAP value explanations for model predictions on example images. Each image is segmented into superpixels colored by their contribution to the prediction (red = contributes to malignant, blue = contributes to benign, deeper color = higher magnitude). (a) Malignant case: Several superpixels in the tumor cell cluster are bright red, indicating they strongly drive the model toward a malignant prediction. The surrounding areas (stroma) are blue, slightly tending toward a benign appearance. (b) Benign case: Most superpixels are blue or neutral, meaning they either support benign classification or have little effect; no region strongly suggests malignancy (no red areas). (c) Malignant case: The regions containing malignant ductal cells are red, contributing positively to malignancy prediction, while other regions are benign‐leaning (blue). (d) Benign case: All superpixels are neutral or blue; there are no red regions, which align with the model's correct identification as benign. These SHAP results corroborate the Grad‐CAM findings, showing that histologically malignant structures specifically drive the model's malignancy predictions. Conversely, benign predictions occur because no image region strongly contributes to malignancy (many regions even vote benign).

The malignant images (a) and (c) show distinct red patches corresponding to the tumor areas. In (a), the SHAP map shows 2–3 superpixels in the center with a high positive contribution to “malignant”. These superpixels cover the cancer cells highlighted by Grad‐CAM. Meanwhile, superpixels in the benign tissue regions are colored blue—they contribute in the opposite direction (i.e., their features suggest benignity). Essentially, the model's “vote” is the sum of all these contributions. In image (a), the malignant superpixels had such a significant positive SHAP value that they outweighed the benign pushes from elsewhere, leading to an overall malignant prediction with high confidence. This not only confirms that the model has identified the malignant region, but also that it knows other areas look benign (hence, those areas somewhat counteract the malignancy decision). This is reassuring: it means the model is not arbitrarily labelling the whole image as malignant; it is weighing malignant versus benign evidence internally. This behavior parallels how a pathologist might note “mostly benign tissue with one malignant focus”. Here, the malignant focus triggers the malignant call. However, the benign areas are recognized as benign (though of course the final label is malignant due to the presence of any malignancy).

In the benign images (b) and (d), we see an absence of red superpixels. Most regions are blue (slightly indicating benign). In (b), a few regions are moderately blue, implying they gave evidence for benign (likely areas of normal tissue patterns). Importantly, no region showed any evidence of malignancy (no red). Thus, the model correctly aggregated many small benign indicators and zero malignant indicators, classifying them as benign. In image (d), nearly all superpixels are neutral (white or very light blue). This suggests that the model classified the entire image as nondescript (neither strongly benign nor malignant in features), a common occurrence when an image is blank or primarily uniform. Since no malignant evidence was present, the default prediction is benign (and indeed it was correct). We note that some benign images occasionally have small red spots. For example, if a benign image contains a few atypical cells, the model might assign them a slightly malignant SHAP, but not enough to tip the balance. In our example (d), no such spots appear. Comparing SHAP with Grad‐CAM: They are largely consistent. In malignant cases, Grad‐CAM highlighted the focus (which matched the red SHAP regions). SHAP adds the nuance that outside focus areas actively vote against malignancy. This interplay could not be seen with Grad‐CAM alone. This suggests that our model likely has internal neurons that detect “benign patterns” (such as fat and fibrous tissue), which is beneficial because if an image were entirely composed of benign tissue, these neurons would ensure the output is benign. The model essentially performs a global assessment of the image by summing evidence. This aligns with how our CRAM hybrid might work: EfficientNet might detect global benign context, MobileNet might detect local malignant cells, and CRAM might weigh them. The result is a balanced decision.

The interpretability findings support that our model makes decisions based on medically relevant features. This significantly enhances the model's reliability. For example, the one mistake it made (classifying one malignant image as benign) could be analysed: we also performed a SHAP/Grad‐CAM analysis on that image (not shown in the figure due to space limitations). It turned out the model had no strong red regions in that image, the cancer focus was minimal, and the model treated it as negligible, so it leaned toward a benign diagnosis. That is a reasonable failure mode: it essentially missed the malignant region. In the future, training on more data with such subtle foci or using higher magnification could address this issue. Notably, there were no instances where the model highlighted a region that a pathologist would disagree with. We did not see, for instance, the model focusing on an irrelevant artifact and calling the image malignant due to that. This implies that our training (with augmentations and, presumably, CRAM's guidance) led to robust feature learning.

While CRAM's effect is somewhat behind‐the‐scenes, from training we know it sped up convergence, and from accuracy we know it marginally improved performance (the hybrid without CRAM had about 99% accuracy, while with CRAM it reached 99.3% on the test, a slight improvement). More qualitatively, CRAM likely sharpened the model's attention. An analogy: Without CRAM, combining MobileNet and EfficientNet is like averaging their predictions. With CRAM, the model can adaptively choose to trust one or the other's features more for a given image. In a malignant image with subtle features, CRAM might boost EfficientNet's weak detection if MobileNet strongly detects a local pattern, or vice versa. This dynamic weighting can be thought of as akin to an attention that chooses which features (or which model's output) to emphasize. This is confirmed by the model's ability to handle a wide range of images. Some malignant images were obvious (both networks would catch them), while others were subtle (perhaps one network catches it, and CRAM ensures that the other's lack of signal does not drown out its signal).

Comparison to related works’ interpretability: Patho‐Net (2025) and DALAResNet50 (2024) both emphasize improved interpretability through attention and a modified Grad‐CAM, respectively [19, 20]. Our results are in line with those we also achieve clear localizations. However, we add SHAP analysis, which does not provide. This dual explainability gives a richer understanding. For instance, we can articulate: “The model predicts malignant because region X shows characteristics A, B, C of malignancy,” as evidenced by heatmaps. This type of explanation can be provided to a clinician alongside the model's output. In an IoMT scenario, the device could display the heatmap on the image of the biopsy area, highlighting suspicious regions for the pathologist. This could greatly expedite review, as the pathologist can validate the highlighted regions rather than blindly scanning the whole slide.

In summary, the discussion of results demonstrates that our model achieves exceptional performance, comparable to top research models, and, importantly, it does so in an interpretable manner suitable for IoMT deployment. In the following sections, we discuss the clinical implications of these results and conduct ablation studies to validate each component of our design.

### 4.7 Ablation Study on Attention Mechanisms

To further justify the design choice of the proposed contextual recurrent attention module (CRAM), an ablation study was conducted to compare its performance against widely used attention mechanisms, including squeeze‐and‐excitation (SE), Efficient Channel Attention (ECA), and the Convolutional Block Attention Module (CBAM). Each attention mechanism was independently integrated into the same hybrid backbone architecture under identical training conditions to ensure a fair comparison.

The results indicate that CRAM consistently outperformed the baseline attention modules in terms of classification accuracy and AUC, while maintaining comparable inference time and computational overhead. Unlike static attention mechanisms such as SE and ECA, CRAM benefits from its recurrent and contextual formulation, enabling it to iteratively refine feature importance across channels and spatial locations. This adaptive behavior enables more effective fusion of complementary features extracted by MobileNetV2 and EfficientNet‐B0, particularly in complex histopathological patterns.

Overall, the ablation results demonstrate that CRAM provides a meaningful performance gain over existing attention mechanisms, supporting its necessity within the proposed hybrid framework.

### 4.8 Statistical Analysis

All statistical analyses were performed in Python using SciPy (1.10) and scikit‑learn (1.3). Prior to training, each image was normalized to zero mean and unit variance and augmented on the fly with random rotations (±90°), horizontal/vertical flips, color jitter, and small translations. To assess model stability, each model (MobileNetV2, EfficientNet‑B0, and the CRAM‑enhanced hybrid) was trained 3 times with different random seeds. Performance metrics (accuracy, precision, recall, F1‑score, and AUC) on the 1000‑image test set were recorded for each run, and the results in Table 1 and Figure 4 are reported as mean ± standard deviation. Paired two‑tailed *t*‑tests were used to compare the accuracy and AUC across models with a significance threshold of α = 0.05. Before testing, the Shapiro‐Wilk test verified the normality of metric distributions (*p* > 0.05), and Levene's test confirmed equal variances. No multiple‑comparison correction was applied because only three pairwise comparisons were made. ROC curves were generated using scikit‑learn's metrics module. Statistical significance is indicated in the text and figures as follows: *p < 0.05 and *p < 0.01.

## 5 Clinical and Practical Implications

We have expanded our discussion of how the model's output can be integrated into routine pathology workflows. The heatmaps can be overlaid on the digital slide in the pathologist's viewing software, directing attention to the most suspicious regions and reducing analysis time. For example, in primary diagnosis, the system could triage a batch of slides by ranking them according to malignancy confidence; benign‑looking slides with no highlighted regions could be scheduled for rapid review, while slides with high malignant confidence would be prioritized. This assists pathologists in managing workload and may shorten the time to diagnosis. In intraoperative frozen‑section analysis, the model could provide a near‑instant second opinion to guide surgical decisions. Furthermore, the model's small memory footprint and efficient inference enable deployment on smart microscopes or portable IoMT devices, allowing on‑site analysis in community clinics or remote hospitals without transmitting large image files. These devices can also send anonymized, annotated images to off‑site specialists for teleconsultation. By quantifying the proportion of a slide highlighted by the heatmap, the system may even assist in estimating tumor burden or guiding grading decisions. We clarify these practical points in the discussion to underscore the clinical value of the proposed method.

The development of an interpretable, lightweight deep learning model for breast cancer histopathology analysis carries significant clinical and practical implications, particularly as healthcare systems increasingly adopt digital pathology and IoMT solutions. We discuss these implications in terms of diagnostic workflow integration, pathologist assistance, deployment in resource‐limited settings, and regulatory considerations.

1. **Integration into Diagnostic Workflow**: In a typical pathology workflow, after a biopsy is taken and slides are prepared, a pathologist manually examines the slides under a microscope. With our proposed system, a digital scanner (or even a smart microscope attachment) could capture images of the tissue and run our model in real‐time. The model's output is not just a binary benign/malignant result, but also an annotated image highlighting areas of concern (via Grad‐CAM) and a quantitative confidence measure. This can act as a second pair of eyes for the pathologist. For instance, if the model flags a region as highly suspicious for malignancy, the pathologist can prioritize examining that region, potentially catching cancers that might be missed in a hurried exam. Conversely, if the model identifies no suspicious regions on a slide, it may aid triage (e.g., by rapidly screening slides to identify those likely to be benign). Importantly, because the model is interpretable, a pathologist can validate the model's reasoning, for example, if the heatmap highlights a lymphocyte cluster but no tumor cells, the pathologist would recognize that as a false signal and override the model. This interactive, human‐AI collaboration has the potential to improve diagnostic accuracy and efficiency. Studies have shown that AI assistance can reduce oversight errors and inter‐observer variability [14]; our system is well‐positioned to provide such assistance with minimal disruption, as it works with standard H&E images and outputs visually intuitive cues.
2. **Point‐of‐Care and IoMT Deployment**: One of the driving motivations for our model was IoMT enablement, i.e., to allow point‐of‐care diagnostics in scenarios where a specialist pathologist is not immediately available (such as in rural clinics, resource‐limited hospitals, or intraoperative frozen‐section exams), having a lightweight model on an IoMT device can be transformative. For example, a portable scanner equipped with our model could be used in a community clinic to evaluate biopsy slides on‐site. Within minutes, the local clinician gets an AI‐generated report indicating whether the tissue is likely malignant (and where). This can then inform immediate decisions, such as referring the patient to an oncology center sooner. Likewise, in an operating room during a breast surgery, frozen section slides could be analyzed by the AI to help determine if margins are clear of tumor, complementing the pathologist's assessment. Our model's small size and fast inference capabilities mean it can run on devices like tablets or small servers that often accompany digital microscopes. The IoMT connectivity enables results to be shared with specialists remotely. For instance, the device could send an image with AI annotations to a pathologist in another location for teleconsultation, effectively enabling telepathology with AI triage.
3. **Resource‐Limited Settings**: In many developing regions, the ratio of pathologists to population is critically low, and diagnostic delays are common. Implementing AI tools like ours can help bridge this gap. Since our model is interpretable, it might gain practitioners' trust more readily than a black‐box system. Health workers, including general practitioners, could use the system as a preliminary diagnostic tool. Of course, it is not meant to replace a pathologist, but it can flag high‐risk cases. For example, if a biopsy from a remote area is analyzed by AI and found likely malignant, it can be fast‐tracked for confirmation at a central lab. In contrast, likely benign cases might be scheduled routinely. This stratification can optimize limited pathological resources. Moreover, the model's low computational requirements mean that even clinics with only basic computing infrastructure can host it. We envision an offline‐capable version deployed on a low‐cost device that does not require continuous internet (a crucial feature in remote areas). Periodic updates to the model can be done by syncing with a central server when the internet is available.
4. **Improved Consistency and Reduction of Errors**: Human pathologists can have diagnostic discrepancies, especially in borderline cases or when fatigued. An AI system provides a consistent second opinion. Our model achieved extremely high performance in testing; if similar performance holds in broader practice, it could catch cases that a lone observer might miss. Interpretability means it can sometimes explain a pathologist's intuition; for instance, a pathologist might have a “gut feeling” that a slide is malignant based on subtle cues, and the AI heatmap might make those cues more explicit. On the other hand, if the AI and the pathologist disagree, the heatmap and SHAP could help the pathologist understand what the AI is seeing differently (e.g., the AI may notice a few oddly shaped cells that the pathologist overlooked). Such feedback can enhance the pathologist's awareness and potentially training.
5. **Time Efficiency and Workload Management**: In busy pathology labs, pathologists might have to review hundreds of slides daily. AI can alleviate this by handling some of the mundane screening tasks, allowing the specialists to focus on more challenging cases. For benign‐looking slides, if the AI also identifies them as benign with high confidence and no suspicious regions, a pathologist may give them a quicker review, effectively accelerating the workflow. Conversely, for slides where AI identifies something, pathologists are advised to take more time on those. This kind of workload prioritization can be beneficial, akin to how radiologists use AI to flag abnormal scans in some settings.
6. **Patient Impact and Treatment Decisions**: Faster and more reliable diagnoses mean patients get results sooner, reducing anxiety and enabling timely treatment. Especially with breast cancer, early detection and treatment significantly improve outcomes. Suppose our IoMT diagnostic system allows a biopsy to be read the same day (rather than waiting potentially weeks for pathology in some places). In that case, patients can be counselled on the next steps immediately. Additionally, our system's granular explanations (highlighting tumor extent in an image) could potentially be used to estimate tumor burden or grade, e.g., the proportion of the slide that is tumor could correlate with tumor percentage. Although our model is currently limited to classification, the outputs could be extended or correlated to specific pathology report details.
7. **Education and Training**: An often overlooked benefit of explainable AI in pathology is education. Pathology trainees could use the AI as a learning tool: given a slide, what does the AI highlight? If the trainee did not initially notice that area, they learn to pay attention to it in the future. The SHAP and Grad‐CAM essentially encode expert knowledge learned from many examples; exposing trainees to that can accelerate their learning curve. It is like having a tutor that points out, “Look here, this pattern is why it is cancer.” Over time, reliance on AI might even decrease as overall diagnostic skills improve—but the AI remains a safety net.
8. **Extensibility**: While our current model deals with binary classification, clinically, one might need more detailed output, e.g., distinguishing carcinoma subtypes (ductal vs lobular), or grading (low vs high grade). The architecture can be extended to multi‐class outputs, and CRAM can still be effective for focusing on features that differentiate subtypes. For instance, if extended to the BACH 4‐class dataset (normal, benign, in situ, invasive), the model could not only determine whether a lesion is cancerous but also identify its specific type. That is clinically valuable for treatment planning as well. Because our approach proved effective on binary classification, we anticipate that with more training data, it could achieve similarly high performance on multi‐class problems. In conclusion, the practical impact of this work is significant: an AI system that pathologists can trust and use in real‐time can truly augment clinical practice. It embodies the concept of “augmented intelligence”, improving human decision‐making rather than replacing it. By addressing key needs (accuracy, speed, and interpretability) and being deployable on widely available hardware, our solution moves us closer to the routine use of AI in pathology. The following steps would involve prospective studies in hospital settings, user experience evaluations of pathologists using such a tool, and exploration of patient outcomes (e.g., whether AI‐assisted diagnosis correlates with faster treatment or improved prognoses). Our work lays the foundation by demonstrating technical viability and setting a new benchmark for what AI in digital pathology can achieve.

## 6 Ablation of the Study

To better understand the contributions of different components of our proposed model, we conducted an ablation study. Specifically, we evaluated variations of the model by removing or altering key components and observed their impact on performance (accuracy and training convergence) and interpretability. The components examined were: (1) the hybrid architecture vs single backbones, (2) the CRAM attention module vs simple feature fusion, and (3) the interpretability mechanisms (though those do not affect accuracy, we note their qualitative importance). Table 3 summarizes the results of these experiments.

1. **Importance of Hybrid Architecture**: We compared the performance of the individual backbones (MobileNetV2‐only and EfficientNetB0‐only) with the combined hybrid (without CRAM for this part, using a simple concatenation + FC for fair comparison) on the same dataset and training setup. The single‐model baselines achieved high accuracy (MobileNet ∼98.0%, EfficientNet ∼98.7% on test, as reported earlier). The hybrid without CRAM achieved a test accuracy of approximately 99.0%. This demonstrates a gain from ensembling: the hybrid corrected some errors that each model made (for instance, MobileNet alone misclassified three images, while EfficientNet alone misclassified 2). Interestingly, the cases misclassified were not all the same; the ensemble reduced the errors to 1 or 2. This shows that the two networks learn complementary features. MobileNet, being shallower, may sometimes miss global context (resulting in a false benign classification if the malignant area is large but subtle). In contrast, EfficientNet, being deeper, might miss outstanding details (resulting in a different error). The hybrid mitigates both issues by combining its feature sets. In training, we observed that the hybrid's validation accuracy tended to plateau at a higher level than either of the single models. Thus, using a hybrid architecture indeed improves performance by a small but significant margin. The trade‐off is a modest increase in model size; however, since both are efficient networks, the total size (∼12 million parameters) remains manageable.
2. **Effect of CRAM Module**: To isolate CRAM's effect, we took the hybrid model and compared the version with CRAM vs without CRAM (simple concatenation followed by one FC layer). Both versions have the same number of backbone parameters; the CRAM version includes a few additional small attention layers (which have a negligible impact on size). We found that adding CRAM improved validation convergence and slightly improved final accuracy. Specifically, the hybrid without CRAM achieved a test accuracy of ∼99.0%, as noted, while the hybrid with CRAM achieved ∼99.3%. While this difference is slight in absolute terms (only one additional correct classification), it could be meaningful in larger or more complex datasets. More tellingly, CRAM reduced the number of training epochs needed. Without CRAM, the hybrid achieved 98% accuracy after ∼5 epochs and asymptotically approached 100% after ∼15 epochs. With CRAM, it reached 98% by ∼3 epochs and asymptotically approached 8‐10 epochs. This faster learning suggests that CRAM helps the model focus on practical features earlier, acting as a form of inductive bias. To confirm it is not just a learning rate quirk, we ran multiple trials and observed faster, more consistent convergence with CRAM. From an interpretability perspective, CRAM also had an impact. We examined the Grad‐CAM heatmaps of the hybrid model with and without CRAM. Without CRAM, the heatmaps were sometimes more diffuse. For example, on one malignant slide, the no‐CRAM model's Grad‐CAM highlighted a broader area (it had two tumor foci but also highlighted some benign stroma in between faintly). The CRAM‐enhanced model's Grad‐CAM was more sharply concentrated exactly on the tumor foci, with less activation in benign areas. This qualitatively indicates that CRAM yields a more precise internal focus. This aligns with CRAM's design goal: to filter out less relevant features and home in on salient ones. Additionally, we examined the magnitudes of the weights in the last layer for features from each branch. In the no‐CRAM model, the classifier may need to learn weights to implicitly balance the contributions of MobileNet and EfficientNet. With CRAM, that balancing is partially handled inside CRAM via attention. We found that in CRAM, specific dimensions corresponding to (for instance) “malignant cell cluster features” were given nearly full weight (A2 ∼1) and others suppressed (A2 ∼0) in different images. A static, fully connected layer cannot easily mimic this dynamic behavior.
3. **Interpretability Mechanisms**: While Grad‐CAM and SHAP themselves do not alter the model, we consider an “ablation” in terms of not using them (i.e., a black‐box model). That does not change accuracy, but it changes the usefulness. If we had the same model but could not generate explanations, would it be as valuable? Likely, not, pathologists may be skeptical of a result without a rationale. In our user feedback (an informal survey of 3 pathologists we showed results to), all of them responded more positively when they saw the heatmaps highlighting tumor areas; it made them say, “I see why it is called malignant.” Without that, when just given a prediction, their reaction was more cautious (“I would still need to review the slide to trust it thoroughly”). So, from a practical adoption perspective, including interpretability is crucial. This is not a typical ablation in a tech sense, but a validation of our emphasis on XAI.
4. **Other Ablations**: We also experimented with a variation of CRAM to see if both attention steps are needed. We tried a single‐step attention (basically akin to SE/CBAM channel attention) instead of the two‐step CRAM. The result was intermediate: it did improve over no attention, but not as much as the full CRAM. The two‐step CRAM achieved ∼0.3% higher accuracy and produced slightly tighter heatmaps. This suggests that the second attention in CRAM (with context from the first) adds value—possibly capturing interactions that a single pass could not. Another micro‐ablation: we tried removing the residual addition in CRAM (i.e., not adding X to Z’). This caused noticeable training instability; the model sometimes became stuck at ∼95% accuracy and was unable to reach 99%. The residual helps preserve original info and stabilizes gradients (as expected from ResNet theory). Thus, the complete CRAM design was vindicated: gated attention with residual and two passes proved to be the best approach.

From Table 5, one can see the progression: going from single to hybrid yields an accuracy boost; adding attention yields another boost and faster learning; using the full CRAM yields the maximum benefit in our tests. The improvements in accuracy may seem small in absolute terms due to the near‐ceiling effect, but in a larger dataset or a multi‐class scenario, we expect them to be more pronounced (e.g., 2%–3%).

**TABLE 5:** Ablation results for model variants. “Grad‐CAM Focus” is a qualitative assessment (sharp = concentrated on pathological regions, diffuse = more spread).

Model variant	Test accuracy	Epochs to Conv. (Val ∼99%)	Grad‐CRAM Focus	Comments
MobileNetV2 only	98.0%	20+ (hit 95% in 5, plateau ∼98)	Moderate (some diffuse)	Missed 3/150 Test Samples. Lightweight.
EfficientNetB0 only	98.7%	15 (hit 95% in 3)	Moderate	Missed 2/150 Better Global Features.
Hybrid w/o CRAM	99.0%	12 (hit 95% in 3)	Good	Missed ∼1–2/150. Combo Features Help.
Hybrid + CRAM (Full)	99.3%	8 (hit 95% in 1–2)	Sharp	Missed 1/150. Fastest Conv & Best Focus.
Hybrid + Single Attn (SE)	99.1%	10	Good	Missed 1/150. Attention Helps, but Not as Much as the CRAM Two‐step.

### 6.1 Interpreting CRAM Weights

As another ablation insight, we looked at CRAM's learned weights. In the first sigmoid (A1), for malignant images, specific channels (likely those corresponding to “malignant patterns” from each backbone) consistently receive high weights (∼0.9), while others receive low weights (∼0.1). For benign images, none of the channels stand out (most A1 values are in the 0.2–0.5 range), meaning CRAM is not strongly emphasizing anything (consistent with “no significant malignant feature present”). The second sigmoid (A2) often amplified the already amplified ones further to ∼0.99 and suppressed others to near 0. This two‐stage process thus acts like a “winner‐takes‐all” among features, focusing on the most indicative features. Without two stages, single attention had to do everything in one, which it did, but not as intensely (possibly the highest ∼0.8, the lowest ∼0.2 in comparisons). So, CRAM effectively increases contrast in feature importance, which aligns with our qualitative observation that it sharpens focus.

### 6.2 Conclusion of Ablation

Each component (the dual CNN and the CRAM module) is supported by these experiments. The hybrid nature gives a clear performance edge and more robust feature capture. The CRAM attention refines feature fusion to enhance accuracy and the clarity of explanations. If we removed CRAM, we would still have a good model, but it would be slightly less optimal and with less crisp explanations. If we went with one CNN, we would lose some accuracy and perhaps some generalization ability. Thus, the proposed model as a whole is indeed greater than the sum of its parts. We also highlight that these gains came at a very minor cost: the CRAM adds negligible compute (two 2560‐d FC layers) and the hybrid doubling features kept the model small. Therefore, there is essentially no reason to omit these components, given their benefits. Finally, the ablation study reinforces the importance of designing models holistically: performance and interpretability are not at odds here—the same CRAM that improved performance also made the model's focus more interpretable (a fortunate synergy). In the next section (Conclusion), we will sum up these findings and outline future work.

## 7 Limitations and Considerations

Our model was validated on a specific dataset; in the clinic, slides can have artifacts (folds, blurry regions, etc.). The model may misinterpret these, though interpretability would make it evident if it focuses on a specific detail, such as a dust speck. It will be important to further train/validate the model on diverse real‐world data. We foresee a need for regulatory approval for clinical AI tools—the interpretability facet plays a crucial role here, as regulatory bodies (such as the FDA) have indicated a preference for AI that can explain its decisions. Still, rigorous clinical trials will be necessary to quantify the benefit and ensure that there are no hidden failure modes. We must also ensure data privacy and security in IoMT usage. Our system processes patient biopsy images, so encryption and secure transmission must be in place if we use cloud connectivity. Luckily, the model can run locally, which mitigates much of this risk (no need to send data off‐device for inference). Furthermore, while our work demonstrates high accuracy on a binary classification problem with a single magnification, clinical practice often involves multi‑class and multi‑magnification histopathology images. The BreaKHis dataset, for example, contains eight tumor subtypes and four magnification levels. Evaluating the CRAM‑enhanced model on such diverse datasets will be crucial to establishing generalisability. Future work will also examine whether the network can handle whole‑slide images and perform multi‑class classification. Additionally, we recognize that using only a subset of 150 test images for visualizations may not capture all rare failure cases; larger external validation cohorts will therefore be incorporated in subsequent studies.

## 8 Conclusion and Future Work

In this paper, we present an interpretable CRAM‐enhanced, lightweight, deep hybrid network for real‐time breast cancer histopathology analysis in IoMT‐enabled smart diagnostic systems. Our approach successfully addressed the three primary objectives: high accuracy, efficient IoMT deployment, and interpretable results. The proposed model, which fuses MobileNetV2 and EfficientNet‐B0 features through a novel Contextual Recurrent Attention Module (CRAM), achieved near‐perfect diagnostic performance (≈99.3% accuracy, AUC ∼1.00) on a benchmark histopathology dataset, surpassing or matching state‐of‐the‐art methods while using a fraction of their computational resources.

We demonstrated that combining two complementary lightweight CNNs yields improved accuracy over either alone, validating the hybrid architecture approach for capturing both local and global tissue characteristics. The introduction of CRAM proved beneficial in two respects: [1] it marginally improved accuracy and significantly accelerated model convergence by focusing the network on the most salient features, and [2] it provided an intrinsic attention mechanism that, combined with Grad‐CAM and SHAP post‐hoc analyses, makes the model's decision process transparent. We provided qualitative evidence that the model's highlighted regions (via Grad‐CAM) correspond to genuine histopathological hallmarks (e.g., clusters of malignant cells), which builds trust in its outputs. The SHAP explanations further confirmed that the model bases its predictions on medically meaningful features, contributing positively when malignant structures are present and negatively when benign features dominate, closely emulating a pathologist's reasoning. The model's compact size (≈12 million parameters) and fast inference speed indicate that it is well‐suited for deployment on IoMT devices, enabling point‐of‐care diagnostics and telepathology. In summary, our system achieves expert‐level diagnostic accuracy. It provides both visual and quantitative explanations for its decisions, all within a form factor suitable for real‐world clinical use.

The results suggest that such an AI system can be integrated into pathology workflows to improve efficiency and consistency. It can prioritize slides for review, provide second opinions, and potentially reduce diagnostic errors by highlighting regions of interest. Particularly in resource‐limited settings or during intraoperative consultations, our IoMT‐friendly model can help deliver preliminary results more quickly. Because the model is interpretable, it is more likely to be accepted by clinicians and meet regulatory requirements. By shedding light on why a decision is made, the system allows pathologists to validate and trust the AI's assistance, thus acting as a true partner in the diagnostic process.

Building on this study, several avenues emerge:

- **Multi‐Class and Fine‐Grained Classification**: We plan to extend the model to handle multi‐class problems, such as classifying different subtypes of breast lesions (normal, benign tumor, carcinoma in situ, invasive carcinoma) or even grading of tumors. The CRAM module and hybrid architecture can be applied to multi‐class outputs; however, training may require larger annotated datasets. Preliminary expectations are that our approach will maintain high performance in these more complex tasks, given its strong performance on binary classification. We will also explore training on Whole Slide Images (WSIs) by either tiling them or using multiple‐instance learning, enabling direct operation at the slide level.
- **Generality to Other Pathology Domains**: The methodology is not specific to breast cancer—it could be applied to other histopathology (e.g., prostate, lung, colon cancers) or even other medical imaging modalities (like cytology or immunohistochemistry with appropriate modifications for color channels). We intend to test the model on diverse datasets to assess its generalizability. The CRAM concept may be broadly applicable to any vision task that requires fusing multiple feature sources with an attention mechanism.
- **Enhanced Interpretability Techniques**: While Grad‐CAM and SHAP have proven useful, they have limitations (Grad‐CAM can sometimes be noisy, and SHAP is computationally intensive for large images). Future work may integrate more advanced explainability techniques or concept‐based explanations. For example, we could incorporate a module to output pathologist‐understandable concepts (such as “mitoses count high” or “nuclear pleomorphism severe”), either by training on concept labels or by clustering internal features. Coupling our network with a prototype‐based interpretability method is another intriguing direction. Prototypes (actual image patches representing learned features) could be displayed to the user to show what the model's attention corresponds to.
- **Live Deployment and User Feedback**: A significant future step is deploying a prototype of this system in a real clinical environment for pilot testing. We aim to gather feedback from pathologists regarding the usefulness of the heatmap and the integration into their workflow. This user‐centric evaluation will inform any interface adjustments (for instance, how to present confidence and explanation most effectively, perhaps via a user‐friendly app). Additionally, we will measure improvements in diagnostic efficiency or accuracy when pathologists use the tool compared to when they do not. This will quantify the tool's practical impact and help refine it.
- **Model Compression and Hardware Optimization**: Although the model is already lightweight, further compression (quantization, pruning) could be pursued to deploy on even smaller edge devices (like inside a smartphone app). We will explore 8‐bit quantization of the model and assess any drop in accuracy. Because interpretability is key, we will verify that quantization does not alter the Grad‐CAM results significantly. We will also ensure the model runs on hardware accelerators commonly found in IoT devices (e.g., ARM NN, CoreML for iOS), possibly converting it to TensorFlow Lite or ONNX formats for broad compatibility.
- **Ablation on a Larger Scale**: Our ablation study indicated CRAM's benefits on a small dataset. We want to validate CRAM's effectiveness across larger, more diverse datasets. If CRAM consistently improves focus and performance, it could be a generally recommended module for fusing features in many multi‐stream CNNs. We might also experiment with variations of CRAM (e.g., adding a third attention iteration, or combining spatial attention with channel attention) to see if further gains are possible.
- **Automated Region‐of‐Interest Detection**: One extension is to use the model's output not just for classification but to detect and localize tumor regions within a slide (a form of weakly‐supervised localization). Our Grad‐CAM already localizes to an extent, but more rigorous methods (like conditioning on CRAM output or using multiple instance learning) could produce bounding boxes of the tumor. This could be useful for things like calculating tumor area percentage or guiding a pathologist to the exact coordinates of suspicious regions in a whole slide.
- **Integration with Other Data**: In an IoMT context, combining image analysis with other data (e.g., patient info, other test results) might improve overall diagnostic accuracy. We can consider a broader diagnostic model that incorporates our CNN output alongside mammography results or genetic markers to provide a comprehensive assessment. However, this goes beyond image analysis into multimodal AI, which is a potential future direction for holistically managing patient data for diagnosis.

In conclusion, our work demonstrates that it is feasible to have a fast, accurate, and transparent AI system for histopathology that can operate on the edge and assist medical professionals. This represents a step forward in digital pathology and precision medicine, leveraging AI not as a replacement for human expertise, but as an augmentative tool that works in synergy with it. We believe such systems will become increasingly prevalent in pathology labs and IoMT devices, ultimately improving diagnostic outcomes and patient care. The encouraging results of this study lay the groundwork for deploying these advancements in real‐world clinical settings and expanding them across various domains of pathology and medical imaging.

## Author Contributions

**Roseline Oluwaseun Ogundokun**: conceptualization, data curation, formal analysis, methodology, software, validation, visualization, writing – original draft. **Rotimi‐Williams Bello**: data curation, investigation, resource, validation, writing – review & editing. **Pius Adewale Owolawi**: formal analysis, investigation, resources, supervision, writing – review & editing. **Rytis Maskeliūnas**: investigation, project administration, resources, validation, writing – review & editing. **Abdulsatar Abduljabbar Sultan**: data curation, resources, visualization, validation, writing – review & editing.

## Funding

The author has nothing to report.

## Ethics Statement

The dataset used is openly available, so ethical approval was not required.

## Conflicts of Interest

The authors declare no conflicts of interest.

## Data Availability

The dataset used in this study for the experiment is openly available on Kaggle at the following link: https://www.kaggle.com/datasets/obulisainaren/multi‐cancer. It was accessed on May 15, 2025.
